# Emerging agents and regimens for AML

**DOI:** 10.1186/s13045-021-01062-w

**Published:** 2021-03-23

**Authors:** Hongtao Liu

**Affiliations:** Section of Hematology/Oncology, Department of Medicine, The University of Chicago Medical Center, 5841 S. Maryland Ave, MC 2115, Chicago, IL 60637-1470 USA

**Keywords:** AML, Targeted therapy, Novel treatment

## Abstract

Until recently, acute myeloid leukemia (AML) patients used to have limited treatment options, depending solely on cytarabine + anthracycline (7 + 3) intensive chemotherapy and hypomethylating agents. Allogeneic stem cell transplantation (Allo-SCT) played an important role to improve the survival of eligible AML patients in the past several decades. The exploration of the genomic and molecular landscape of AML, identification of mutations associated with the pathogenesis of AML, and the understanding of the mechanisms of resistance to treatment from excellent translational research helped to expand the treatment options of AML quickly in the past few years, resulting in noteworthy breakthroughs and FDA approvals of new therapeutic treatments in AML patients. Targeted therapies and combinations of different classes of therapeutic agents to overcome treatment resistance further expanded the treatment options and improved survival. Immunotherapy, including antibody-based treatment, inhibition of immune negative regulators, and possible CAR T cells might further expand the therapeutic armamentarium for AML. This review is intended to summarize the recent developments in the treatment of AML.

## Introduction

AML is a heterogeneous disease, defined by a broad spectrum of genomic changes and molecular mutations that influence clinical outcomes and provide potential targets for drug development. The updated 2017 European LeukemiaNet (ELN) risk stratification guidelines combining cytogenetic abnormalities and genetic mutations have been widely used to predict the prognosis of AML patients [1], while others have been exploring to incorporate additional prognostic factors into ELN-2017 guidelines to improve the risk stratification models [2].

Advanced by basic and translational research, especially through large scale genomic analysis to understand the molecular landscape of AML, the development of targeted therapies, such as targeting fms-like tyrosine kinase 3 (FLT3) and isocitrate dehydrogenase 1 and 2 (IDH1 and IDH2) mutations, the treatment of AML landscape changed significantly with FDA approvals for several new drugs in the past several years. Even with all these improvements, primary resistance to initial treatment and disease relapse remain huge unmet need in the treatment of AML. The majority of AML patients still eventually succumb to the disease. We still have a long way to further improve the survival of the AML patients, thus many investigational drugs have been explored to target the primary and secondary treatment resistance in AML patients.

This review will provide updates of the emerging therapeutic approaches for the treatment of AML, including combinations with mutation driven targeted treatments, novel immunotherapies in the myeloid disease.

## Targeted therapies: alone or combination

### BCL-2 inhibitor: venetoclax

BCL-2 is a member of the BCL-2 family of anti- and pro-apoptotic proteins. BCL-2 protects cells against apoptosis. BCL-2 expression in AML has been associated with decreased sensitivity to cytotoxic chemotherapy and a higher rate of relapse [3]. Venetoclax is an orally bioavailable selective inhibitor of BCL-2, promoting intrinsic apoptotic pathway activation resulting in mitochondrial outer membrane permeability through dissociation of BCL-2 mediated sequestration of BH3 proteins BIM and BID and effector proteins BAX and BAK. Venetoclax was initially approved by U. S. Food and Drug Administration (FDA) in 2016 to treat individuals with chronic lymphocytic leukemia (CLL) with deletion (17p).

### *Venetoclax* + *hypomethylating agents or low dose cytarabine*

Early studies using venetoclax as monotherapy in AML demonstrated only modest efficacy in high-risk relapsed/refractory (R/R) AML patients with an overall response rate (ORR) of 38% and complete remission/complete remission with incomplete hematologic recovery (CR/CRi) of 19%. The responses were short lived, with overall survival (OS) of only 4.7 months [4]. Based on promising results from two large Phase 1b/II trials using combination of a hypomethylating agent (HMA) or low-dose cytarabine (LDAC) with venetoclax in untreated older AML patients [5, 6], FDA granted accelerated approval to venetoclax in combination with azacitidine (AZA) or decitabine (DEC) or LDAC for the treatment of newly-diagnosed (ND) AML in adults who are age 75 years or older, or who have comorbidities that preclude use of intensive induction chemotherapies in 2018.

Recently published Phase III randomized studies confirmed the results from these early single arm trials, and demonstrated a significant survival benefit from adding venetoclax to azacitidine and to LDAC [7, 8]. The major findings from the VIALE-A and VIALE-C trials are summarized in Table 1. In summary, the VIALE-A trial included 431 patients without history of exposure to azacitidine. At a median follow-up of 20.5 months, the median OS was 14.7 months in the azacitidine-venetoclax group and 9.6 months in the control group. The incidence of CR and composite complete remission rate (cCR) (CR + CRi) were significantly higher with azacitidine-venetoclax than with the control regimen. However, there were higher rates in key adverse events in the azacitidine-venetoclax group than those in the control group, but they were manageable [7]. The VIALE-C study assigned 211 patients to either venetoclax (*n* = 143) or placebo (*n* = 68) in 28-day cycles, plus LDAC on days 1 to 10. In contrast to VIALE-A trial, 20% enrolled patients had received prior HMA treatments. The planned primary analysis showed a 25% reduction in risk of death with venetoclax plus LDAC *vs* LDAC alone, although this was not statistically significant. Median OS was 7.2 *vs* 4.1 months, respectively. Unplanned analyses with an additional 6-months follow-up demonstrated median OS of 8.4 months for the venetoclax arm (HR, 0.70; 95% CI, 0.50–0.98; *P* = 0.04). CR/CRi rates were 48% and 13% for venetoclax plus LDAC and LDAC alone, respectively. Thus, venetoclax plus LDAC demonstrated clinically meaningful improvement in remission rate and OS *vs* LDAC alone, with a manageable safety profile [8]. Based on these confirmatory data, FDA granted full approval to these venetoclax combinations for treating newly diagnosed AML patients. Both trials established new standard of care for unfit newly diagnosed AML patients. Since VIALE-A trial excluded patients with previous exposure to azacitidine, and 20% patients enrolled on the VIALE-C trial had exposure to HMA, venetoclax plus LDAC might be a preferred consideration for patients who received HMAs in the past.

**Table 1:** Comparison of randomized prospective studies on venetoclax-based combinations in AML: AZA + venetoclax *vs* LDAC + venetoclax

Regimen	AZA + venetoclax	LDAC + venetoclax
Phase	III VIALE-A trial	III VIALE-C trial
Population	Age > 75 years or unfit for chemotherapy	
Control arm	AZA	LDAC
h/o HMA	No	Yes, allowed (20%)
Patient number	431 (286 in AZA + venetoclax)	211 (143 in LDAC + venetoclax)
Median age (range), years	76 (49–91)	76 (36–93)
30-day mortality, %	7%	13%
cCR (CR) rate, %	66.4% (36.7%)	48% (27%)
MRD negativity, %	N/A	6%
Time to CR (response)	1.3 months (0.6–9.9)	N/A most response at the end of cycle 2
Median DOR, months	17.5 (13.6 to NR)	NA
Median OS, months	14.7 (11.9–18.7)	8.4 (5.9–10.1)
Reference	[7]	[8]

Both trials also identified that patients with NPM1 and IDH1/2 mutations had high CR rates of 91%, and 71%, respectively with HMA + venetoclax [5] and high CR/CRi rates (89% and 72%), respectively, when treated with venetoclax + LDAC [6]. Patients with FLT3 mutations (Internal tandem duplication (ITD) and/or tyrosine kinase domain (TKD) also demonstrated high CR rate of 72% [5]. On the other hand, inhibitors to these mutations have been developed and will be discussed in the following sections. It would be continued debate on how to choose the first line treatment for AML with these mutations: hypomethylating agents with IDH1/2 inhibitors *vs* venetoclax-based combination; how to sequence the treatment options: venetoclax-based combinations first followed by IDH1/2 inhibitors at disease relapse/ progression or the other way around; or use three drugs combination with HMA + venetoclax + IDH1/2 inhibitor to get deeper remission. Only randomized clinical trials could eventually answer these important clinical questions.

### *Venetoclax* + *intensive chemotherapy*

Not surprisingly, venetoclax has been studied in combinations with intensive chemotherapy as well (summarized in Table 2). A retrospective report of 13 patients treated with FLAVIDA salvage therapy (fludarabine, cytarabine, and idarubicin in combination with venetoclax 100 mg daily for 7 days; dose reduced due to concurrent azole administration) compared to a control cohort received FLA-Ida (fludarabine, cytarabine, and idarubicin) reported a higher but not statistically significant CR/CRi rate of 69% compared to 47% in the control cohort [9]. A phase 1b/II trial of medically fit patients with R/R AML receiving FLAG-Ida induction and consolidation in combination with a 14 days course of venetoclax was conducted at MD Anderson. Early results were promising with CRc of 74% in all the patients and an impressive CRc of 91% in newly diagnosed (ND) patients. Consistent with known venetoclax resistance mechanisms, high levels of MCL-1 expression were found in patients who relapsed following FLAG-Ida + venetoclax [10]. The updated data of 62 patients (27 with ND AML and 35 with R/R AML) from the trial was recently presented. The ORR was 84%, with 89% of ND AML and 66% of R/R AML patients achieving a CRc. 83% of patients achieved minimal residual disease (MRD) negative (MRD-) status assessed by flow cytometry. After a median follow up of 11 months, median OS was not reached. The addition of venetoclax to FLAG-ida demonstrated robust efficacy with acceptable safety profile [11].

**Table 2:** Summary of venetoclax-based combinations in AML

Combination	Phase	Disease status	Patient number	CR/CRi rate, %	References
FLA-Ida	Retrospective	R/R AML	13	69%	[9]
FLAG-ida	Ib/II	ND AML R/R AML	27 35	89% in ND AML 66% in R/R AML	[10, 11]
CAVEAT (5 + 2)	Ib	ND AML	51	72% in all 97% in de novo AML 43% secondary AML	[12]
DEC10	II	ND AML R/R AML	70 55	86% in ND AML 42% in R/R AML	[13]
CLIA	II	ND AML	18	88%	[14]
CLAD/LDAC, alternating with AZA	II	ND AML	48	94%	[15]
CPX-351	II	R/R AML ND AML	17 1	37%	[16]
CPX-351 LIT	Ib	ND AML	44 planned	NA	[17]
GO	Ib	R/R AML	24 planned	NA	[18]

The CAVEAT study reported data on 51 newly diagnosed patients with AML, either de novo or secondary, who were treated in five venetoclax dose-escalation cohorts (50–600 mg; venetoclax was given over 14 days, day -6 to 7 with induction chemotherapy (cytarabine 100 mg/m^2^ days 1–5 and idarubicin 12 mg/m^2^ intravenously days 2–3)). The same venetoclax dose and schedule was given for four cycles of consolidation (cytarabine, days 1–2, and idarubicin, day 1), and as maintenance (up to seven 28-day cycles). The overall CR/CRi rate was 72%, but was 97% in the 28 patients with de novo AML and only 43% in secondary AML. [12]. In our center, we have used HiDAC + mitoxantrone + venetoclax for several heavily pretreated patients with R/R acute leukemia to control the disease prior to allogeneic stem cell transplantation (allo-SCT) (personal experience). This combination warrants further study in both newly diagnosed and R/R AML setting.

The results of ten-days of decitabine (DEC10) with venetoclax (DEC10-VEN) in AML and high-risk MDS were reported. DEC10-VEN is safe and highly effective in newly diagnosed AML and can serve as an effective bridge to SCT. Median OS in treatment naïve AML patients who subsequently underwent SCT was not reached (1 year OS of 100%). For previously treated AML patients, OS was 22.1 months [13]. In addition, propensity score matched analysis (PSMA) was employed to compare outcomes of 54 younger adult patients with R/R AML treated on the prospective phase 2 trial of 10-day decitabine and venetoclax (DEC10-VEN) with a historical cohort of patients treated with intensive chemotherapy. The analysis demonstrated that DEC10-VEN provided comparable response of CR/CRi, OS, and rate of patient to proceed SCT to non-venetoclax based intensive chemotherapy. Thus, DEC10-VEN represents an appropriate salvage therapy, and provides an appropriate backbone for adding novel therapies in R/R AML patients [19].

The addition of venetoclax to cladribine, idarubicin, and Ara C (CLIA) was safe and effective in ND patients with AML. The combination was not associated with early mortality or prolonged myelosuppression, but did result in high rates of durable MRD negative remissions (NCT02115295) [14]. Addition of venetoclax to a low-intensity backbone of cladribine + LDAC (CLAD/LDAC) alternating with HMA for older patients with newly diagnosed AML provided a CR/CRi rate of 94%; and among the subset of patients who had CR with complete count recovery, the MRD negative rate was 92%. The regimen was well tolerated, with 4-week mortality rates of 0%. With a median follow-up of more than 11 months, the median OS has not been reached (NR), with 12-month OS rates of 70% [15]. Full dose CPX-351 plus 7 days of VEN (300 mg on D2-8) was demonstrated to be tolerable with acceptable toxicities in patients with R/R AML with an ORR of 44%; and ORR was high at 60% in patient without prior VEN exposure, compared to just 17% among those who had prior VEN. 86% of responding patients proceeded to SCT. The median OS overall was 6.4 months; and the median OS was not reached among the responders [16].

Other ongoing trials include open-label, multicenter, 2-part, phase 1b study (NCT04038437) to determine the maximum tolerated dose and evaluate the safety, efficacy, and pharmacokinetics of CPX-351 lower-intensity therapy (LIT) plus venetoclax [17]. Another single arm, open-label, multi-center, dose-escalation phase Ib study is evaluating the combination of venetoclax and gemtuzumab ozogamicin in R/R CD33 + AML patients (NCT04070768) [18].

### *Venetoclax* + *experimental drugs or targeted inhibitors*

Given the proven synergies of BCL-2 inhibition, multiple combinations with targeted agents, and venetoclax are under investigation. There are many ongoing combinations of therapies targeting BCL-2 and other pathways, including FLT3 inhibitors (gilteritinib) and IDH1 and 2 inhibitors (Ivosidenib and enasidenib) (will be discussed in the later sections), MCL-1 inhibitors (VU661013, A-1210477); MEK1/2 inhibitor (cobimetinib), and MDM2 inhibitor (idasanutlin) (reviewed in [20]), combination with TKI in Ph + acute leukemia [21] and other emerging pre-clinical combinations including small-molecule inhibitors of CDK9 (the orally active A-1592668 and the related analog A-1467729) leading to down-expression of MCL-1 [22]; the Exportin inhibitor, Selinexor, [23]; BET inhibitors, ABBV-075, [24]; SRC family kinases (SFK) and Bruton's tyrosine kinase (BTK) inhibitor, ArQule 531 (ARQ 531), [25]; and it is expecting much more novel combinations to come.

### Resistance mechanisms

HMA + venetoclax or LDAC + venetoclax have clearly advanced the treatment of AML for older or unfit AML patients. Unfortunately, these regimens are unlikely to provide cure as most patients have relapsed at the median of 7 cycles of treatment. A retrospective study demonstrated that the outcome of 41 patients who failed to respond to HMA + venetoclax was very poor with the median OS of only 2.4 months despite salvage therapy [26]. To understand the resistance mechanisms, DiNardo CD et al. analyzed 81 patients receiving these venetoclax-based combinations to identify molecular correlates of durable remission, initial response followed by relapse (adaptive resistance), or refractory disease (primary resistance). Acquisition or enrichment of clones with activation of the signaling pathways such as FLT3 or RAS or bi-allelic mutations perturbing TP53 were most commonly identified among primary and adaptive resistance to venetoclax-based combinations. Single-cell studies identified heterogeneous and sometimes divergent interval changes in leukemic clones within a single cycle of therapy, highlighting the dynamic and rapid occurrence of therapeutic selection in AML. In functional studies, gain of FLT3-ITD mutation or loss of TP53 conferred cross-resistance to both venetoclax and cytotoxic-based therapies [27]. These data confirmed the previous findings that TP53 apoptotic network is the primary mediator of resistance to BCL-2 inhibition in AML cells [28]. Interestingly, recent study demonstrated that monocytic AML is intrinsically resistant to venetoclax + AZA due to loss of expression of the venetoclax target of BCL-2, but instead preferentially reliant on MCL-1 for the survival. Thus, venetoclax + AZA treatment selects monocytic disease at disease relapse, which is derived from pre-existing monocytic subclones [29]. AML patients with monocytic disease or TP53 mutation might have high risk to be resistant to venetoclax-based combinations, and clinical trials targeting TP53 mutation or trials specifically targeting monocytic AML might be considered over venetoclax-based combinations.

Future clinical research will focus on deepening the responses provided by HMA + venetoclax with additional targeted agents, like ivosidenib in IDH1 mutated AML (to be discussed in next section), FLT3 inhibitors, and novel pathways inhibitors to eventually cure a greater fraction of newly diagnosed AML, and to explore new strategies to deal with relapses after venetoclax-based therapies.

### IDH1/2 inhibitors

IDH1 and IDH2 are critical enzymes for the oxidative carboxylation of isocitrate. A mutation in one of these genes results in increased concentration of 2-hydroxyglutarate (2-HG). 2-HG causes DNA and histone hypermethylation, leading to blocked cellular differentiation and tumorigenesis. Mutations in IDH1 or IDH2 are present in 5% to 15% and 10% to 15% of patients with newly diagnosed AML, respectively [30]. Oral, small-molecule inhibitors have been developed for both mutant IDH1 (ivosidenib) and IDH2 (enasidenib). In R/R AML, ivosidenib and enasidenib as single agent produced promising responses for the corresponding mutations with ORR of 41.6% (CR: 21.6%) with median OS of 8.8 months [31] and ORR of 40.3% (CR 20.6%) with median OS of 9.3 months [32] respectively. FDA approved ivosidenib and enasidenib for patients with relapsed or refractory IDH1 and IDH2 mutated AML, respectively, in 2018. In the front line setting, both inhibitors have also demonstrated clinical effectiveness [33, 34], leading to FDA approval of ivosidenib for patients with newly diagnosed IDH1 mutated AML based on an ORR of 42% (CR: 30%) with median OS of 12.6 months in older patients not eligible for intensive therapy [34].

The Phase 3 IDHENTIFY study evaluating enasidenib plus best supportive care (BSC) versus conventional care regimens, which included BSC only, azacitidine plus BSC, low-dose cytarabine plus BSC, or intermediate-dose cytarabine plus BSC, did not meet the primary endpoint of OS in patients with R/R AML with an IDH2 mutation. The safety profile of enasidenib was consistent with previously reported findings. IDH inhibitors alone are unlikely to provide cure or durable remission for R/R AML, but they might provide excellent disease control with low toxicity and a bridge to allo-SCT.

IDH inhibitors work in part through induction of differentiation of malignant cells, leading to differentiation syndrome in 10% to 20% of patients. Clinical features are similar to those seen in patients with acute promyelocytic leukemia (APL) treated with ATRA-based regimens [35, 36]. Early studies established a firm association between IDH mutations and serum 2-HG concentration in AML, and confirmed that serum oncometabolite measurements provide useful diagnostic and prognostic information that can improve patient selection for IDH-targeted therapies [37]. However, 2-HG level reduction and clearance of IDH mutation by next generation sequencing (NGS) assay does not correlate with the clinical response. These inhibitors are unlikely to provide cure of the AML due to primary resistance from co-mutations in other pathways especially the NRAS/KRAS, and MAPK pathway effectors PTPN11, NF1, FLT3 and others [38] and secondary resistance from development of second-site IDH2 missense mutations or isoform switching [39, 40].

Since IDH1/2 mutations lead to DNA and histone hypermethylation, HMAs might have synergistic effects in combination of IDH inhibitors. Combination of HMAs with IDH inhibitors has been studied. The combination of ivosidenib and azacitidine was studied in 23 patients with IDH1 mutated AML as front line treatment. The ORR was 78% with CR/CRh rate of 70%, and median time to response of 1.8 months; median response duration was not yet reached. The ivosidenib and azacitidine combination was well tolerated with a safety profile consistent with ivosidenib or AZA monotherapy and with 17% incidence of IDH differentiation syndrome. Clearance of mutated IDH1 was seen in 63% patients with CR/CRh. CR and ORR rates exceeded those expected from AZA alone [41]; 83% CR/CRh patients achieved MRD negativity by flow cytometry [42]. AGILE, a global, double-blind, randomized, placebo-controlled, phase III trial for patients with previously untreated IDH1 mutated AML who are not candidates for intensive therapy (NCT03173248) is actively enrolling patients from 172 study centers across the world [43]. Patients are randomly assigned to AZA + ivosidenib or AZA + placebo.

As for the IDH2 inhibitor of enasidenib, the phase II portion of an open-label, randomized phase I/II study of enasidenib (E) + AZA (“E + A”) *vs* AZA monotherapy (“A”) in patients with mutated IDH2 (mIDH2) ND AML (NCT02677922) was recently reported [44]. 101 patients with intermediate- or poor-risk cytogenetics were randomized 2:1 to E + A or A in 28-day cycles. ORR (71% *vs* 42%) and CR (53% *vs* 12%) rates were significantly improved with E + A with greater clearance of mIDH2 allele frequency. Time to first response was about 2 months in each arm and the time to CR was 5.5 months (range, 0.7–19.5). There was no difference in median PFS and OS so far [44].

As discussed in the section of Azacitidine and venetoclax, this combination is very effective in patients with IDH1/2 mutation. In a pooled retrospective study, 79 patients with IDH1/2 mutation were identified and treated with VEN + AZA on either the Phase Ib or the randomized Phase III (VIALE-A) trials. CR/CRh was 72% (95% CI: 61%-82%) in the whole population. In patients with IDH1, CR/CRh was 59%, median time to first CR/CRh response was 2.3 months, and median duration of response (DOR) and OS were 21.9 (7.8–29.5) months and NR. In patients with IDH2, CR/CRh rates were 80%, median time to first CR/CRh response was 1.0 month. Median DOR and median OS (mOS) were NR. Thus, VEN + AZA provided high response rates, long DOR, and mOS among treatment-naïve patients with IDH1/2 mutation ineligible for intensive chemotherapy with acceptable safety profile [45]. As mentioned previously, it will be a continued debate to optimize the front line treatment for unfit AML patients with IDH1/2 mutations.

There is also a rationale for combining IDH inhibitors with BCL-2 inhibitors, since the accumulation of 2-HG caused by IDH mutations could decrease the mitochondrial threshold for induction of apoptosis induced by BCL-2 inhibition with venetoclax [46]. The combination therapy of ivosidenib (IVO) plus venetoclax (VEN) with or without azacitidine was found to be effective against AML harboring an IDH1 mutation in a phase Ib/II trial [47]. Patients with AML or high-risk MDS were assigned to one of three cohorts, either receiving IVO + VEN 400 mg, IVO + VEN 800 mg, or IVO + VEN 400 mg + AZA. The median time to best response was 2 months. In 18 evaluable patients, cCR rate was 78% overall (treatment naive: 100%; R/R: 75%), and 67%, 100%, and 67% by cohort with median time to best response of 2 months. IVO + VEN + AZA therapy was well tolerated and highly effective for patients with IDH1 mutated AML [47]. It is reasonable to expect that combination of the IDH2 inhibitor enasidenib with venetoclax and azacitidine might also provide better outcomes than enasidenib alone or enasidenib with azacitidine, since enasidenib plus venetoclax demonstrated superior anti-leukemic activity against IDH2 mutated AML in patient-derived xenograft models [48]. Table 3 summarizes the trials of combinational therapies for newly diagnosed unfit AML patients with IDH1/2 mutation.

**Table 3:** Summary of combination of targeted-therapy trials in IDH1/2-mutant newly diagnosed AML

Regimens	Phase	Patient Number	CR/CRi rate, %	Time to CR or response (median), months	OS (median), months	References
HMA + venetoclax	Ib	35	71	2.1	24.4	[5]
AZA + venetoclax	III	46	75.4	N/A	N/A	[7]
AZA + venetoclax	Pooled data from two trials	79	72	1.0	24.5	[45]
LDAC + venetoclax (HMA naïve)	Ib/II III	18	72	1.4	19.4	[6, 8]
AZA + ivosidenib	Ib	23	69.6	3.7	N/A	[49]
AZA + enasidenib	II	68	68	5	22.0	[50]
Venetoclax + ivosidenib	Ib/II	12	83	NA	NA	[47]
AZA + venetoclax + ivosidenib	Ib/II	6	67	NA	NA	[47]

### Targeting FLT3 mutations

FLT3 Mutations occurs in approximately 30% patients with newly diagnosed AML (20% to 25% with FLT3-ITD mutation, 5% to 10% with FLT3-TKD), and associates with more proliferative disease, increased risk of relapse, and inferior survival. Randomized phase III RATIFY study led to approval of 7 + 3 + midostaurin as front line for young fit patients [51], and randomized phase III ADMIRAL study with single-agent gilteritinib established the approval of gilteritinib for the treatment of R/R FLT3-mutated AML [52]. The phase III randomized trial using quizartinib *vs* investigator choice salvage chemotherapy in patients with R/R FLT3-ITD mutated AML met the primary objective of OS improvement [53], leading its approval in Japan, but not in the US. Many studies to combine these FLT3 inhibitors are under investigation.

An open-label, phase 1 study (NCT02236013) assessed the safety/tolerability and anti-leukemic effects of gilteritinib plus 7 + 3 induction, consolidation, and maintenance therapy in fit adults with newly diagnosed FLT3-mutated AML. 80 patients with median age of 59 years were allocated to treatment. The maximum tolerated dose of gilteritinib was 120 mg daily. CRc was achieved by 81.8% of patients across all dose groups with mutational clearance (FLT3 ITD signal ratio of ≤ 10^–4^ after induction or consolidation) was achieved by 70% of patients with FLT-ITD mutation receiving a gilteritinib dose of ≥ 120 mg [54]. Two large randomized clinical trials of induction and consolidation chemotherapy plus gilteritinib *vs* midostaurin in FLT3 mutated AML patients are ongoing in the US (PrECOG trial) (NCT03836209)) and in Europe (HOVON 156 AML / AMLSG 28–18 trial (NCT04027309)).

The LACEWING study is a phase 3 trial to randomize FLT3 mutated ND AML patients ineligible for intensive induction chemotherapy to get gilteritinib plus azacitidine *vs* azacitidine alone. The safety cohort enrolled 15 patients and established dose of gilteritinib of 120 mg to be used in combination with azacitidine. Overall, a CRc of 67% was observed with median duration of remission of 10.4 months for the CRc responders. The combination treatment was well tolerated with no unexpected adverse effect [55]. While the data provides a promising option of gilteritinib plus azacitidine for newly diagnosed FLT3-mutated unfit AML patients, the company announced that Phase 3 LACEWING trial failed to meet primary end point of OS at a planned interim analysis and the study was terminated for futility in December 2020. Many lessons have been learned in the AML field that high response rate in AML will not necessary transform into survival benefit.

In the R/R setting, a phase 1b study tested the safety and efficacy of combining venetoclax at 400 mg with gilteritinib at 120 mg daily. 39 patients were enrolled, and among them 64% had previous history of FLT3 TKI exposure. 37 patients were evaluable for response, 31 (84%) achieved CRc. This data compares favorably to the CRc of 54% with single agent gilteritinib in the ADMIRAL study; suggesting gilteritinib plus venetoclax might be better option for R/R FLT3-mutated AML, while longer follow-up with OS data is awaited [56]. A Phase Ib/II trial explored the combination of quizartinib (Quiz) with decitabine (10 days) ± venetoclax mostly in patients with R/R AML. CRc of 90% was achieved in the DEC10 + VEN + Quiz cohort, and CRc rate of 40% was achieved in DEC10 + quiz cohort. In addition, CyTOF (single-cell mass cytometry) analysis could be used to select patients with the best response based on pre- and on-therapy apoptotic and signaling pathway profiles [57].

### Targeting TP53 mutation

The *TP53 gene,* located on chromosome 17p13.1, is commonly mutated in tumors making it one of the most widely mutated genes in human malignancies. *TP53* mutations are detected in 5% to 20% of patients with newly diagnosed AML and MDS, with higher incidences in older patients and in those with secondary AML or therapy-related myeloid neoplasms. *TP53* mutation is enriched in patients with complex karyotype and monosomal karyotypes and also in patients with relapse or refractory disease. *TP53* mutation has been associated with a poor prognosis in both AML and MDS [58, 59].

A recent study analyzed 3,324 patients with MDS for *TP53* mutations and allelic imbalances, and delineated two subsets of patients with distinct phenotypes and outcomes. One-third of *TP53*-mutated patients had monoallelic mutations whereas two-thirds had multiple hits consistent with biallelic targeting. Established associations with complex karyotype, few co-occurring mutations, high-risk presentation and poor outcomes were specific to multi-hit patients only. The *TP53* multi-hit state predicted a high risk of death and leukemic transformation independently of the revised international prognostic scoring system (IPSS-R). Importantly, monoallelic patients did not differ from *TP53* wild-type patients in outcomes and response to therapy. This study demonstrates that consideration of *TP53* allelic state is critical for diagnostic and prognostic precision in MDS as well as for future correlative studies of treatment response [60]. It is uncertain if a similar finding will be identified in AML patients.

TP53 mutation used to be considered as “undruggable”, but there has been an abundant effort recently to explore different mechanisms to overcome the negative impact of the mutant TP53 protein. Although one study using 10-days of decitabine reported a marrow remission rate of 100% in TP53-mutated patients with AML or MDS [61], the results have not been confirmed in subsequent studies, including a randomized study of 5-day versus 10-day schedules of decitabine as first line therapy for older patients with AML [62].

APR-246 (Eprenetapopt) is a novel, first-in-class small molecule that selectively induces apoptosis in TP53 mutated cancer cells via thermodynamic stabilization of the TP53 protein and shifting the equilibrium toward the wild-type conformation with restoration of the transcriptional activity of unfolded wild-type or mutant *TP53* [63]. Updated results from the multicenter Phase 1b/2 trial demonstrated that APR-246 + AZA is a well-tolerated combination with high response rates in HMA-treatment naïve TP53 mutated higher risk MDS, MDS/MPN, and oligoblastic AML (20%-30% blasts) patients (NCT03072043). Patients enrolled on the Phase II portion received APR-246 4500 mg IV (days 1–4) + AZA 75 mg/m2 SC/IV × 7 days (days 4–10 or 4–5 and 8–12) in 28 day cycles. 55 patients were enrolled, and ORR by IWG criteria was 87% with CR of 53%. Median time to response was 2.1 months, and median duration of response of 6.5 months. CR rate was 50% for AML. An isolated TP53 mutation was predictive for a higher CR rate (69% *vs* 25%; *P* = 0.006) with a trend for higher ORR. By intention-to-treat analysis, median OS was 11.6 months (95% CI 9.2–14) with significantly longer OS in responding patients (12.8 *vs* 3.9 months; *P* < 0001). The study concluded that APR-246 + AZA is a well-tolerated combination with high response rates in TP53 mutated MDS/AML. Response durations were promising and were accompanied by a high fraction of cytogenetic and deep molecular remissions leading to encouraging outcomes post-SCT. These data support the ongoing, randomized phase 3 study of APR-246 + AZA versus AZA alone in TP53 mutated MDS (NCT03745716) [64]. A similar phase 2 study conducted by the Groupe Francophone Des Myelodysplasies (GFM) in a high-risk elderly population of TP53 mutated MDS and AML patients reported response rate of 76%, including 53% CR/CRi [65].

Beyond *TP53* mutation or loss, MDM2 forms a complex with wild type TP53, leading to decreased *TP53* transcriptional activity, increased nuclear export, and degradation of TP53 through the proteasome. Inactivation of wild-type TP53 protein frequently occurs in the cancer cells through overexpression of its negative regulator MDM2. Thus, MDM2 antagonists have been explored to re-establish the function of wild TP53, and various compounds have been developed to disrupt this MDM2–TP53 interaction [66]. These MDM2 inhibitors could synergistically activate the TP53 pathways with various chemotherapy to kill leukemia cells, but this class of drugs will be largely ineffective in *TP53*-mutated disease [67].

Several MDM2 inhibitors are being evaluated in patients with AML/MDS [68]. Nutlins were the first small molecule inhibitors developed that bind to MDM2 and target its interaction with TP53 [69]. Second-generation nutlin, such as idasanutlin, have improved potency and better toxicity profile. Data from early phase trials demonstrated clinical response with monotherapy with idasanutlin (RG7388) or in combination with other agents [68]. In general, monotherapy with MDM2 inhibitors revealed very modest anti-leukemia effect in R/R AML, including RG7112 [70], RO6839921 (an inactive pegylated prodrug of idasanutlin) [71], and AMG-232 [72]. Other MDM2 inhibitors under investigation pre-clinically or in early phase clinical trials were reviewed [73]. Currently, efforts are focusing on combination strategies. In a multicenter Phase 1/1b study, idasanutlin in combination with cytarabine resulted in cCR of 29% with CR rate of 25% in the dose escalation, dose expansion, and bridging cohorts. The median duration of response was about 6.4 months (1.1–11.9 months) and some patients remained in CR at 1 year follow-up. Higher MDM2 expression in leukemic blasts and stem cells, and not TP53 mutational status was associated with CR, suggesting MDM2 expression in leukemic cells might serve as a predictive biomarker for response [74]. Unfortunately, even with the promising results from the early phase, MIRROS trial (NCT02545283) [75], a randomized Phase III trial evaluating idasanutlin + cytarabine versus placebo + cytarabine in R/R AML was terminated due to failure to meet its primary goal of prolonging the survival, further demonstrating the challenges to replicate the early promising data at the later large phase trial in AML patients.

Earlier studies demonstrated the cross-talk between TP53 pathway and apoptosis-related molecules, initially with BCL-2 and BAX [76–81], and later with MCL-1 [82]. There was synergistic apoptosis-induction effects in leukemia cells with TP53 activation following suppression of pro-apoptotic molecules, like BCL-2, BCL-XL and XIAP [82, 83]. More recent studies clearly demonstrate that TP53 apoptotic network is a primary mediator of resistance to BCL-2 inhibition in AML cells [28], and increased activities of TP53 through MDM2 inhibition negatively regulates the RAS/RAF/MEK/ERK pathway and activates GSK3 to modulate MCL-1 phosphorylation and promote its degradation, thus overcoming AML resistance to BCL-2 inhibition by venetoclax [84]. The combination of venetoclax with the MDM2 inhibitor, idasanutlin, has been tested in a phase Ib study in older patients with relapsed or refractory AML [85]. The response rates were promising with ORR of 37% across all dose cohorts, and an ORR of 50% in the dose cohorts being considered for recommended Phase II dose (RP2D). MRD negativity (< 0.1% by Flow) was achieved in 43% of patients with cCR. The median time to response was 1.8 months (range, 0.8–2.7), with median response duration of 8.1 months (range, 0.3–9.7). Median overall survival in the RP2D cohorts was 5.3 months (range, 0.2–17.6). The response rate was very high at 86% in patients with IDH2 mutation and 57% in patients with a RUNX1 mutation, but only 20% in patients with TP53 mutation [85].

MDM2 antagonists have been combined with other agents in different cancer types, such as with PI3K, MEK, or FLT3-ITD pathway inhibition in AML, with CD20 antibody in lymphoma, with CDK4/6 inhibitor in locally advanced or metastatic liposarcoma, with PD-L1/PD-1 antibodies in patients with metastatic solid tumors, and a number of others [73].

## Immunotherapies

In some ways, immunotherapy was not novel for the treatment of AML, since the graft *vs* leukemia (GVL) effect had been well documented in allo-SCT and donor lymphocyte infusion (DLI), and allo-SCT had driven the improved survival of AML patients in the past several decades prior to the advent of the novel agents. Compared to the rapid development of immunotherapies, especially checkpoint inhibitors using PD-1 and PD-L1 antibodies, in solid tumors, even with clear early scientific support of the PD-1-PD-LI pathway in the pathogenesis of AML, immunotherapy in AML has been lagging far behind solid tumors. In addition, lacking unique targets such as CD19 and CD22 in lymphoid leukemia/lymphoma to make CAR T therapies successful in lymphoid diseases, we still have a long way to develop cellular therapies for myeloid leukemia. In this section, the available data on checkpoint inhibitors, antibody-based treatments and cellular therapies under investigation for myeloid malignancies will be discussed (summarized in Table 4).

**Table 4:** Immunotherapies in AML

Modality	Targets	Agents	Clinical setting	Efficacy	Reference
Checkpoint inhibitors	CTLA-4	Ipilimumab	Relapse after SCT	CR in 4/12 with extramedullary relapse	[86]
	PD-1/PD-L1	Nivolumab	R/R AML	ORR of 33%	[87]
		Durvalumab	ND AML	No difference between Durvalumab + AZA vs AZA alone	[88]
		Nivolumab	maintenance	Promising from single arm Phase II Randomized Phase II in CR1 is pending	[89]
Macrophage “Do not eat me”	CD47	Magrolimab	ND AML	ORR: 65% ORR: 71% for TP53 mutated AML	[90]
Leukemia stem cell	Tim-3	MBG453 + HMA	ND AML R/R AML	With AZA: ORR: 29% for ND and R/R With Decitabine: ORR: 41% for ND ORR: 24% R/R	[91]
ADC	CD123	IMGN632	R/R AML	ORR: 18%	[92]
BiTE/DART	CD123	Flotetuzumab	R/R AML	ORR: 42%	[93]
		Vibecotamab	R/R AML	ORR: 15%; 26% with low burden disease	[94]
CAR T	Various targets: CD33, CD123,	All in the early phase	R/R AML	Too early to tell	NA

### Checkpoint inhibitors

Up-regulation of negative T cell co-stimulatory receptors, such as CTLA-4 and PD-1, on tumor-specific T cells to inhibit their effector function has been well studied in solid malignancies, and recent advances in immunotherapy led to the approval of PD-1 and PD-L1 antibodies in multiple cancer types and cancers with certain features. In pre-clinical AML models, blocking CTLA-4 in combination with a peptide-based vaccine led to enhanced CTL responses and prolonged survival, providing rationale for targeting this receptor in AML patients. With regard to the PD-1/PD-L1 pathway, pre-clinical data clearly demonstrated that this pathway is also involved in immune evasion in AML [95–97]. In addition, T cells from the patients with AML have increased expression of inhibitory checkpoint molecules including PD-1, Tim-3, and Lag3 compared to T cells from healthy donors, contributing to immune exhaustion and possible AML disease relapse [98]. Hypomethylating agents up-regulate the expression of checkpoint pathways, including PD-1, PD-L1 and CTLA-4 in myeloid diseases [99]. Up-regulation of other checkpoint pathways, like Gal9/Tim-3, might also contribute to the resistance of AML to chemotherapy [100].

Checkpoint inhibitors have been explored in myeloid disease. An early study using anti-CTLA4 antibody, ipilimumab, as a single agent in patients with hematologic malignancies who relapsed after allogeneic SCT, demonstrated modest efficacy in myeloid diseases, including 4 durable CR in patients with extramedullary disease relapse among 12 patients with AML [86]. In contrast, single agent PD-1 inhibition by nivolumab had no response in R/R AML, while the combination of nivolumab with HMA, azacitidine, generated ORR of 33% (22% CR + CRi). The ORR of 58% in HMA-naïve relapse/refractory AML patients compared favorably with the historical control using azacitidine alone [87]. The study also demonstrated that pre-treatment bone marrow CD3 and CD8 counts were significantly predictive for response. Thus, these could be used to select patients for better response, and future trials might focus on HMA-naïve, first relapse AML patients. As expected, immune mediated toxicities remain major concern, and about 25% of patients developed grade 2–4 immune toxicities; nivolumab immune-related adverse events led to treatment discontinuation in nearly 1 in 7 patients. These results need to be confirmed in large randomized trial. Based on promising single center Phase II data, the leukemia intergroup designed and activated the randomized phase III trial (SWOG 1612, NCT03092674) in December 2017 to evaluate the combination of azacitidine with or without nivolumab as front line therapy in older patients with AML and MDS-RAEB2. Results were reported at the ASH 2019 meeting after 113 patients were screened and 78 randomized to study treatment. The trial experienced two challenges: (1) the required administration of 7-days of azacitidine at the enrolling sites created a burden for this population and (2) there was an excess of early deaths in the azacitidine/nivolumab arm compared with the control arm. Thus, the trial has been placed on hold since October 2018 [101].

The field had another set back from the data presented at 2019 ASH meeting using the PD-L1 inhibitor, durvalumab, in combination of azacitidine as the front line treatment for older patients with AML who were unfit for intensive chemotherapy and for patients with HR-MDS. This large, international, randomized Phase 2 study enrolled a total of 213 patients, 84 with MDS and 129 with AML, but had negative results [88]. As expected, immune-mediated AEs were observed, but all were manageable and resolved, and reassuringly, there were no new safety signals or potential overlapping risks identified with the combination. While up-regulation of PD-L1 by azacitidine was confirmed, there was no treatment-mediated induction of PD-L1 surface expression observed on myeloid blasts, and there was no clinically meaningful difference in efficacies observed between treatments for either cohort, further suggesting that we still have a long way to go to use biomarker driven patient selection to identify the patients who might benefit from checkpoint inhibitor alone or in combination with chemotherapy.

For AML patients who are not candidates for SCT, the standard of care was observation after induction/consolidation chemotherapy prior to the recent FDA approval of oral azacitidine for maintenance. Unfortunately, more than 50% of patients eventually experience a disease relapse. The outcome of older AML patients (> = 60 years) is poor with an estimated 2 year PFS of only 20% without SCT. Thus, new post-remission strategies are needed in order to improve the long-term outcomes of AML patients. The PD-1 antibody, nivolumab has been studied as maintenance post-chemotherapy in AML patients. A pilot phase II study of nivolumab maintenance in high risk AML patients in CR, ineligible for SCT was reported at 2018 ASCO meeting. 14 patients including 11 in CR1, 2 in CR2, and one in CR4 were enrolled and treated. Therapy was well tolerated, although 5 patients (36%) had grade 3/4 immune-related events. 4 patients could continue treatment after interruption, and one was taken off the study. This small study reported 1-year CR duration and OS estimates of 71% and 86%, respectively [89]. Through the NCI ETCTN, a randomized phase II trial to evaluate maintenance nivolumab versus observation in patients with AML in first CR/CRi after induction and consolidation (NCT02275533) reached full enrollment at the end of 2019. The results are eagerly awaited.

### Targeting CD47 pathway

In addition to checkpoint inhibitors in T cells, immunotherapy now includes inhibition of macrophage’s “Do not eat me” signal on cancer cells, mainly through the interaction of CD47 on the tumor cells with signal-regulatory protein *α* (SIRP*α*) on the macrophages. CD47 was shown to be overexpressed in myeloid malignancies, leading to tumor evasion of phagocytosis by macrophages, and blockade of CD47 leads to engulfment of leukemic cells and therapeutic elimination [102]. Supported by pre-clinical data demonstrating robust anti-cancer activity in AML and MDS by anti-CD47 antibody, clinical trials using Hu5F9-G4 (magrolimab), in combination with azacitidine demonstrated exciting results in both intermediate to very high risk MDS patients and in untreated AML patients. These data were presented, and included TP53 mutated AML patients [103]. The trial utilized a magrolimab priming/intra-patient dose escalation regimen (1–30 mg/kg weekly) to mitigate on target anemia. Magrolimab + AZA was well-tolerated with a safety profile similar to AZA monotherapy. Initial data indicate that magrolimab may be particularly effective in TP53 mutant patients, a treatment-refractory subgroup. Magrolimab has been awarded breakthrough status by the FDA. The results in AML patients were recently updated. Fifty-two AML patients were treated with magrolimab + AZA. Thirty-four patients were evaluable for efficacies. Of these patients, 65% achieved an objective response with CR/CRi rate of 56% (44% + 12%). Median time to response was 2.04 months. The median OS for TP53 wild-type AML patients was 18.9 months. In patients with TP53-mutant AML, the response rate was 71% with CR/CRi rate of 67%. The median OS was 12.9 months. Expansion cohorts in TP53-mutated AML are ongoing (NCT03248479) and a phase 3 trial evaluating magrolimab + AZA in untreated TP53-mutant AML patients is planned [90]. Patients with newly diagnosed TP53 mutated AML should be referred for these trials.

### Targeting Tim-3 pathway

T-cell immunoglobulin and mucin domain-containing 3 (Tim-3) is a negative regulator of T cells. Tim-3 was initially described as an inhibitory protein expressed on interferon-gamma secreting activated T cells. Recent studies have confirmed that Tim-3 participates in multiple co-inhibitory receptors and contributes to the dysfunctional or ‘exhausted’ T cells in chronic viral infections and cancer. Furthermore, co-blockade of Tim-3 and PD-1 can improve anticancer T cell responses in patients with advanced cancers [104]. Furthermore, Tim-3 has also been identified as an antigen on AML stem cells that is also present on leukemic blasts but not normal hematopoietic stem cells. Anti-Tim-3 antibody treatment has shown efficacy in blocking engraftment of AML in a mouse xenotransplantation model [105].

MBG453 (Sabatolimab) is a high-affinity, ligand-blocking, humanized anti-Tim-3 IgG4 antibody which blocks the binding of Tim-3 to phosphatidylserine (PtdSer). A phase Ib study of the MBG453 in combination with decitabine in patients with high-risk MDS (HR-MDS) and AML was recently presented [106]. The combination was tolerable and no study treatment-related deaths were observed. Clinical efficacy with MBG453 in combination with decitabine had been seen at doses ranging from 240 mg Q2W to 800 mg Q4W. 8 of 16 (50%) HR-MDS patients achieved modified CR or CR, 4 of 14 (29%) newly diagnosed AML patients had achieved a response of PR or better (2 PR, 2 CR), and 5 of 17 (29%) R/R AML patients had achieved a response of CRi. Median onset of response among all patients was 2.0 months. Tim-3 expression was detected on leukemic cells, with modulation of Tim-3 expression following treatment with decitabine. These findings validate Tim-3 as a promising therapeutic target in MDS and AML [106].The Phase I trial of the combination of MBG453 with decitabine or azacitidine reported the outcomes of 106 patients with high or very high-risk MDS (*n* = 34) or ND or R/R AML (*n* = 72). All patients were naïve to HMAs and were considered poor candidates for intensive chemotherapy. Patients received escalating doses of intravenous MBG453 240 mg or 400 mg once every 2 weeks (days 8 and 22) or 800 mg once every 4 weeks (day 8), plus intravenous decitabine 20 mg/m2 on days 1–5 or subcutaneous or intravenous azacitidine 75 mg/m^2^ on days 1–7, every 28 days. The response rates for evaluable patients in the decitabine and azacitidine arms appeared promising. For MBG453 + Decitabine, ORR among evaluable patients was 58% for HR-MDS and 41% for ND-AML; and 24% for R/R-AML. Median exposure duration among responders was 8.6 (2.0–26.7) months. For MBG453 + AZA, with relatively short follow-up (median exposure duration, 3.0 months), ORR among evaluable patients was 70% (7/10) for HR-MDS and 27% (3/11) for ND-AML. Responses were observed across all three MBG453 dose levels with both combinations. Based on the PK/PD modeling, MBG453 400 mg Q2W and 800 mg Q4W were predicted to have similar average steady state PK concentrations and similarly high receptor occupancy rates (> 95% occupancy in 95% of patients) [91]. The results for 48 ND AML were updated at ASH 2020, the combination of HMAs + Sabatolimab was tolerable with most commonly reported AEs infrequent and consistent with those reported for patients treated with HMA alone, only 6.3% patients discontinued treatment due to AEs. The ORR was 41% with median time to response at 2.1 months; and 78.8% patients had response after 6 month with 12 months PFS rate of 32.2% [107]. These data demonstrated HMA + sabatolimab was well tolerated with promising anti-leukemia activity, providing a new platform for future improvement. Later phase trials are ongoing including a phase II multi-center, single arm, safety and efficacy study of MBG453 in combination with azacitidine and venetoclax for the treatment of AML in adult patients unfit for chemotherapy (STIMULUS-AML1 (NCT04150029)).

## Antibody-based treatments

Antibody-based treatment plays an important role in lymphoid disease, including naked antibodies rituximab and obinutuzumab targeting CD20; antibody–drug conjugates (ADC) such as brentuximab and inotuzumab ozogamicin; T cell-redirecting antibody, such as the first and only approved bispecific T-cell engager (BiTE) blinatumomab. However, antibody-based therapies in myeloid leukemia have been limited to gemtuzumab ozogamicin (GO), an ADC combining a recombinant IgG4 humanized mAb against CD33 that is conjugated to calicheamicin, a potent DNA damaging toxin. After an uneven drug development course, GO has been re-approved for front line use in combination with standard induction therapy for AML patients with favorable or intermediate risk cytogenetics, and as a single agent for older patients who are unfit for intensive chemotherapy, or in patients with relapsed/refractory AML. Based on the clinical efficacy of GO, several other CD33 antibody constructs have been developed in AML, but non-specific killing of CD33-expressing hematopoietic stem cells contributed to excessive hematologic toxicities, leading to the termination of the front line phase III CASCADE study of azacitidine with or without vadastuximab (conjugated to pyrrolobenzodiazepine dimer) in older patients with AML [108].

JNJ-67571244, is a novel human bispecific antibody capable of binding to the C2 domain of CD33 and to CD3, capable to induce T-cell recruitment and CD33 + tumor cell cytotoxicity. JNJ-67571244 demonstrated good in vitro cytotoxicity of CD33 + AML cell lines and also exhibited significant antitumor activity in vivo in mouse models of human AML. JNJ-67571244 is currently in a phase 1 clinical trial in patients with relapsed/refractory AML and high-risk myelodysplastic syndrome (#NCT03915379) [109].

Beyond targeting CD33, CD123 has been a molecule of interest in both AML and blastic plasmacytoid dendritic cell neoplasm (BPDCN). CD123, also known as interleukin-3 receptor alpha (IL-3R*α*), is a type I transmembrane glycoprotein, which is strongly expressed on AML leukemic blasts and leukemic stem cells, but only at a very low level on normal hematopoietic stem cells, thus making CD123 a promising therapeutic target. ADC against CD123, tagraxofusp (SL-401; Elzonris) received FDA approval in 2018, to treat adults and children 2 years of age and older BPDCN patients based on very high ORR of 90% [110]. Other CD123 ADCs under investigation include IMGN632, a conjugate of a novel CD123-targeting antibody with a highly potent DNA alkylating payload. IMGN632 is active in preclinical models of AML at concentrations far below levels that impact normal bone marrow cells. Importantly, IMGN632 exerted a potent anti-leukemic effect in various AML xenograft models, strongly supporting the clinical development of IMGN632 [111]. IMGN362 was studied in a phase I evaluation for relapsed/refractory CD123-positive hematological malignancies (NCT 03386513). The most common treatment-related adverse events were infusion-related reactions, which were manageable with dexamethasone prophylaxis. No cytokine release syndrome (CRS) had been observed so far. Overall, 13 of 71 (18%) patients with AML who received the ADC in the trial obtained a CR/CRi. Responses were observed across the range of patients with AML including those patients with relapsed/refractory disease, those with relapsed/refractory de novo AML, those with adverse cytogenetics as defined by European LeukemiaNet criteria, and those with prior SCT [92]. Another trial is exploring the combination of IMGN362 with HMA or venetoclax (NCT04086264) [112], since synergistic anti-leukemia effect has been reported [113].

T cell re-directing antibodies are also in development targeting CD123, MGD006 is a novel CD3-CD123 dual-affinity re-targeting antibody (DART), developed by MacroGenics (Rockville, MD, USA). It exhibited a potent anti-leukemic activity both in vitro and in vivo and was well tolerated in an animal model [114]. MDG-006, with the generic name of flotetuzumab has been evaluated in phase I/II clinical study in refractory/relapsing AML patients. Among the primary induction failure/early relapse patients treated at RP2D of 500 ng/kg per day, the CR/CRh rate was 26.7%, and ORR rate was 30.0%. Infusion-related reaction/cytokine release syndrome occurred frequently from 81 to 100% in different cohorts, but grade 3 or above only happened in 3.3–8.0% patients, and was managed with standard supportive care [115]. The outcomes of 38 patients with primary induction failure or early relapse (PIF/ER) treated on the trial was recently presented, the overall complete response rate (CRR) was 42.1% with 68.8% subsequently undergoing allo-SCT. Median time to first response was 1 cycle. With a median follow up time of 10.8 months, median OS was 4.5 months. No grade ≥ 3 CRS events had been reported in this cohort. Most CRS events (51.5%) occurred in the first week of treatment during step-up dosing, the incidence of CRS progressively decreased during dosing at RP2D [93]. Enrollment to this study is ongoing to better define biomarkers to predict response and identify patients more likely to respond to this drug.

A Phase I clinical trial studied the bispecific monoclonal antibody, XmAb14045 (Vibecotamab), targeting both CD123 and CD3 in patients with CD123 expressing leukemia, mainly R/R AML. The trial demonstrated evidence of anti-leukemic activity as a single agent in heavily pretreated patients with R/R AML with a 23% CR/CRi rate [116]. The study is ongoing with further dose escalation cohorts, and the updated data was recently presented. 112 patients with R/R AML had received vibecotamab. Patients were heavily pretreated with a median of three prior therapies and 30% patients with a history of allo-SCT. CRS was the most common toxicity occurring in 61% of patients, and 9% of patients experienced CRS at grade 3 or higher. The majority of CRS was observed in the first dose and was generally manageable with premedication. The ORR was 15% in 54 evaluable patients who received a dose of at least 0.75 mcg/kg. The ORR increased to 26% for patients with a baseline blast count less than or equal to 25% in the bone marrow, suggesting vibecotamab might provide better efficacy in patients with low burden of disease [94].

In addition to using antibodies against CD3 to target T cells, there has also been an effort to target NK T cells through CD16 to explore its anti-leukemia effect, such as a dual-targeting triplebody 33–16-123 (SPM-2) agent, targeting CD33 and CD123, and using CD16 to engage NK cells. The cytolytic activity of NK cells mediated by SPM-2 was demonstrated in vitro using primary leukemic cells from 29 patients with a broad range of AML-subtypes. Maximum susceptibility was observed for leukemic cells with high combined density of CD33 and CD123 above 10,000 copies/cell [117]. It now needs to be assessed in the early phase clinical trials in real patients.

## CAR T cells treatment

Chimeric antigen receptor (CAR) T-cell therapies have been very successful in lymphoid hematologic malignancies, such as ALL, diffuse large B-cell lymphoma, and multiple myeloma due to unique targets such as CD19, CD22, and BCMA. CAR T cells treatment in myeloid hematologic malignancies had been challenging due to lack of authentic AML-specific surface antigens, in order to avoid targeted killing of normal hematopoietic cells. Early CAR T treatments have been mainly focusing on CD33, and CD123 as discussed in the antibody-based treatment, while other potential targets, like CLL1 [118], FLT3, NKG2D, Lewis Y, CD44v6, CD38, CD7 et al. [119], and LILRB4 in monocytic AML [120] have been explored. Several strategies have been developed to optimize the efficacy and safety profile of CAR T-cell therapies in AML, including “suicide switch” control of the CAR T cells using inducible caspase-9 and modification of the affinity of the CAR T cells to target only cells with high target expression [121]. So far, only very limited clinical data have been presented in myeloid disease, mainly CD33 and CD123 based CAR T cells. To date, only a single case of a patient with R/R AML treated with anti-CD33 CAR T cells (CART-33) has been reported. The patient experienced symptoms of CRS within 2 weeks of the infusion of CAR T cells, and had marked decrease of blasts in the bone marrow at the same time, but the response was transient and there was florid disease progression at 9 weeks after the cell infusion [122]. In addition, a compound CAR (cCAR) (CLL1-CD33 cCAR) comprising an anti-CLL1 CAR linked to an anti-CD33 CAR via a self-cleaving P2A peptide and expressing both functional CAR molecules on the surface of a T-cell were engineered and tested. An alemtuzumab safety switch to CD52 was also established to ensure the elimination of CAR T cells following tumor eradication. The CLL1-CD33 cCAR demonstrated anti-leukemic activity in AML cell lines, primary human AML samples, and multiple mouse models, and phase I clinical trial has been ongoing [123]. Investigators presented the latest updates of the trial recently. Between January 2018 and September 2019, 9 R/R AML patients were enrolled (NCT03795779). CRS occurred in 8 patients (3 grade I, 3 grade II, and 2 grade III). Neurotoxicity occurred in 4 patients (1 grade I and 3 grade III). All CRS and neurotoxicity resolved after treatment, and it was proposed that early intervention with steroids could significantly reduce CRS and neurotoxicity. Disease reevaluation 4 weeks post CAR T cell infusion revealed 7 of 9 patients were MRD- by flow cytometry. 2 of 9 had no response, one of which was CD33 + /CLL1-, indicating the importance of CLL1 targeting in this CAR T cell treatment. For the 7 patients who reached MRD-, 6 patients moved to a subsequent allo-SCT mostly with RIC conditioning. Five patients successfully engrafted with persistent full chimerism. One died of sepsis on day + 6 before engraftment [124].

CAR T cell trials against CD123 have been started, including using autologous or allogeneic T cells [125, 126]. CD123 CAR T clearly have anti-leukemia effects in vitro using leukemia cell lines or primary patient leukemia cells, and in vivo using leukemia mouse models. However, it is still controversial if CD123 CAR T cell could markedly suppress normal hematopoiesis [127, 128].

## Oral azacitidine (CC-486, onureg) in maintenance

Disease relapse has remained as the main issue for the treatment of AML and MDS, and even after allo-SCT. Targeted treatment with FLT3 inhibitor, midostaurin, has been given with induction and consolidation, then maintenance [51]. Sorafenib maintenance was recently demonstrated to reduce the risk of relapse and death after allo-SCT for FLT3-ITD-positive AML in the randomized clinical trial, providing the first solid evidence for the success of maintenance post allo-SCT in AML patients [129], and Phase III MORPHO Trial is under way to evaluate gilteritinib as maintenance after allo-SCT in patients with FLT3 mutation-positive AML (NCT02997202). R/R AML with IDH1 and IDH2 mutations have been treated with IDH1/2 inhibitors, the treatment with IDH1/2 inhibitors will continue even after reaching beneficial response if not moving to allo-SCT could be counted as maintenance therapy in some way.

Oral azacitidine, CC-486 (brand name of Onureg), became the first FDA approved drug specifically as maintenance treatment for AML patients in CR1 based on the positive survival results of the QUAZAR AML-001 maintenance trial [130]. In the trial*,* between May 2013 and October 2017, 472 patients with de novo or secondary AML with intermediate- or poor-risk cytogenetics were enrolled after achieving first CR or CRi after intensive chemotherapy and received 0–2 courses of consolidation chemotherapy. They were not candidates for SCT. Patients were randomized 1:1 to receive CC-486 300 mg or placebo (PBO) once-daily on days 1–14 of repeated 28-day treatment cycles starting within 4 months of attaining CR/CRi. At a median follow-up of 41.2 months, OS was significantly improved with CC-486. Median OS was 24.7 months *vs* 14.8 months from time of randomization, respectively (*P* = 0.0009; HR 0.69 [95% CI 0.55, 0.86]). RFS was also significantly prolonged: median RFS was 10.2 months in the CC-486 arm, compared with 4.8 months in the PBO arm (*P* = 0.0001; HR 0.65 [95% CI 0.52, 0.81]). The OS and RFS benefits of CC-486 were demonstrated regardless of baseline cytogenetic risk, the number of prior consolidation cycles received, and CR *vs* CRi status. CC-486 had a manageable safety profile generally consistent with that of injectable azacitidine. CC-486 is the first therapy used in the maintenance setting to provide statistically significant and clinically meaningful improvements in both OS and RFS in patients with AML. Subgroup analysis of the patients enrolled on the QUAZAR AML-001 trial demonstrated CC-486 improved OS and RFS regardless of the number of consolidation cycles (0, 1, or ≥ 2 cycle consolidation) received prior to study entry. These data clearly suggest that older patients with AML in CR1 after induction can benefit from oral azacitidine maintenance, regardless of their fitness to receive consolidation or the number of consolidation cycles received before starting maintenance [131]. In addition, while MRD + status at the entry of the study was associated with shorter OS and PFS comparing to patients with MRD- status, CC-486 maintenance improved OS and RFS independent of MRD status, and provided higher rate of conversion from MRD + at the beginning of the treatment to MRD- on the study at 37% comparing to only 19% for patients on placebo [132]. Furthermore, QUAZAR AML-001 trial captured patient-reported health-related quality of life (HRQoL) assessed using two validated instruments, the Functional Assessment of Chronic Illness Therapy (FACIT) Fatigue Scale and the European Quality of Life–Five Dimensions–Three Levels (EQ-5D-3L) questionnaire. There was no clinically meaningful differences occurred between CC-486 and placebo during the treatment course by FACIT-Fatigue scores, two significant differences observed in EQ-5D-3L scores between treatment arms at cycles 22 and 23 were not clinically meaningful and likely due to chance. Thus, oral azacitidine significantly improved OS and RFS without compromising HRQoL comparable to placebo [133]. These analysis further advocate the benefit of oral azacitidine maintenance for AML patients in CR1.

On the other hand, a retrospective study evaluated the clinical outcomes in patients enrolled in QUAZAR AML-001 who relapsed with 5–15% blasts on-study who then received escalated 21-day dosing of study drug. 91 patients from both arms were identified as having early AML relapse with 5–15% blasts and were assigned to receive a 21-day/cycle dosing schedule. Among 78 evaluable patients, 23% of patients in the CC-486 arm and 11% of patients in the placebo arm regained CR/CRi while receiving an escalated dosing regimen with well tolerated toxicities. Thus, a 21-day CC-486 dosing schedule could be considered for patients who experience AML relapse with ≤ 15% blasts on the maintenance [134].

It will require additional studies to see if oral azacitidine alone or with other agents like venetoclax could replace IV or SC azacitidine in therapeutic areas other than for maintenance of AML.

## Summary

With many new treatment options for AML, we are entering into a new era to decide the choice and sequence of treatment options for AML patients. A brief road map of AML treatments is included in Table 5. Even with all these progress, the treatments of AML still have huge unmet needs, thus it is paramount to refer AML patients at the initial diagnosis and at the time of relapse for available clinical trials if possible. Combination therapies, especially HMAs with single or multiple targeted therapies will continue to dominate the treatment landscape of AML in the near future. Hopefully, immunotherapy might eventually play a larger role in AML treatment. Elimination of MRD with immunotherapy, targeted therapy, or the combination might provide the ultimate cure for some AML patients with or without allo-SCT. It is important to continue to enroll AML patients in well designed, rigorous science-driven clinical trials. With the help from basic and translational research, additional targets could be identified and real personalized therapies could be achieved based on the genomic and molecular features of the disease in individual AML patient.

**Table 5:** Roadmap of AML treatments

ND AML	IC candidate	FLT3 mutation	7 + 3 + midostaurin Clinical trial: 7 + 3 + midostaurin *vs* 7 + 3 + gilteritinib
		Low, Intermediate risk (CD33 +)	7 + 3 + GO
		Secondary AML	CPX351
		IDH1/2 mutation	7 + 3 or clinical trial
		No targetable Mutation	Clinical trial or 7 + 3 like regimens
		TP53 mutation	Clinical trial or HMA-based regimens
	Not IC candidate	IDH1/IDH2 mutation	Clinical trial HMA + venetoclax LDAC + venetoclax AZA + IDH inhibitor
		FLT3 mutation	Clinical trial HMA + venetoclax LDAC + venetoclax HMA + gilteritinib
		No mutation	Clinical trial HMA + venetoclax LDAC + venetoclax
		TP53 mutation	Clinical trails AZA + magrolimab AZA + APR-246 Off trial: HMA + venetoclax LDAC + venetoclax 5 day or 10 day Decitabine
R/R AML		IC candidate	Re-induction best on the clinical trial
		CD33 +	Clinical trials or GO based regimens,
		IDH1/2 mutation	Ivosidenib/enasidenib alone or HMA combination Venetoclax-based combinations (2 drugs or 3 drugs)
		FLT3 mutation	Gilteritinib alone or combinations with HMA Venetoclax-based combination, (2 drugs or 3 drugs)
		NPM1 mutation or MLL rearrangement	Clinical trials with NPM1/MLL inhibitors Venetoclax-based combination, (2 drugs or 3 drugs)
		TP53 mutation	Clinical trials
		No mutation	Clinical trials Novel first in class agents HMA-base combinations Immunotherapy: MoAbs ADC BiTE/DART Cellular therapies, CAR T, NK cells…

## Conclusion

The landscape of the treatment of AML changed dramatically in the past several years with multiple new FDA approved drugs. The outcomes for most AML patients still remain dismay and well-designed clinical trials with robust patient participation are the key to future success.

## Data Availability

Not applicable.

## Abbreviations

Allo-SCT: Allogeneic hematopoietic stem cell transplantation
AML: Acute myeloid leukemia
BSC: Best supportive care
CI: Confidence interval
CIR: Cumulative incidence of relapse
CR: Complete remission
CRc: Composite CR (includes CR, CRi, and CRp)
CRi: CR with incomplete hematologic recovery
CR_MRD_: CR with absence of minimal residual disease
CRp: CR with incomplete platelet counts
DFS: Disease-free survival
EFS: Event-free survival
FDA: US Food and Drug Administration
HR: Hazard ratio
KM: Kaplan-Meier
MDS: Myelodysplastic syndrome
MLFS: Morphological leukemia-free state
MRD: Minimal (measurable) residual disease
ND: Newly diagnosed
OS: Overall survival
RFS: Relapse-free survival
SCT: Stem cell transplantation
TTE: Time-to-event
APL: Acute promyelocytic leukemia
ATRA: All-trans retinoic acid
CLL: Chronic lymphocytic leukemia
mCR: Bone marrow CR
HiDAC: High dose cytarabine (AraC)
HMA: Hypomethylating agent
LDAC: Low dose cytarabine
ITD: Internal tandem duplication
TKD: Tyrosine kinase domain
ORR: Overall response rate
RP2D: Recommended phase 2 dose
CLIA: Cladribine, idarubicin, and AraC
R/R: Refractory/relapsed
BiTE: Bispecific T cell engager
DART: Dual-affinity Re-targeting antibody

## References

[1] Döhner H, Estey E, Grimwade D, Amadori S, Appelbaum FR, Büchner T, Dombret H, Ebert BL, Fenaux P, Larson RA (2017). Diagnosis and management of AML in adults: 2017 ELN recommendations from an international expert panel. Blood.

[2] Pogosova-Agadjanyan EL, Moseley A, Othus M, Appelbaum FR, Chauncey TR, Chen IML, Erba HP, Godwin JE, Jenkins IC, Fang M (2020). AML risk stratification models utilizing ELN-2017 guidelines and additional prognostic factors: a SWOG report. Biomark Res.

[3] Campos L, Rouault JP, Sabido O, Oriol P, Roubi N, Vasselon C, Archimbaud E, Magaud JP, Guyotat D (1993). High expression of bcl-2 protein in acute myeloid leukemia cells is associated with poor response to chemotherapy. Blood.

[4] Konopleva M, Pollyea DA, Potluri J, Chyla B, Hogdal L, Busman T, McKeegan E, Salem AH, Zhu M, Ricker JL (2016). Efficacy and biological correlates of response in a phase II study of venetoclax monotherapy in patients with acute myelogenous leukemia. Cancer Discov.

[5] DiNardo CD, Pratz K, Pullarkat V, Jonas BA, Arellano M, Becker PS, Frankfurt O, Konopleva M, Wei AH, Kantarjian HM (2018). Venetoclax combined with decitabine or azacitidine in treatment-naive, elderly patients with acute myeloid leukemia. Blood.

[6] Wei AH, Strickland SA, Hou JZ, Fiedler W, Lin TL, Walter RB, Enjeti A, Tiong IS, Savona M, Lee S (2019). Venetoclax combined with low-dose cytarabine for previously untreated patients with acute myeloid leukemia: results from a phase Ib/II study. J Clin Oncol.

[7] DiNardo CD, Jonas BA, Pullarkat V, Thirman MJ, Garcia JS, Wei AH, Konopleva M, Dohner H, Letai A, Fenaux P (2020). Azacitidine and venetoclax in previously untreated acute myeloid leukemia. N Engl J Med.

[8] Wei AH, Montesinos P, Ivanov V, DiNardo CD, Novak J, Laribi K, Kim I, Stevens DA, Fiedler W, Pagoni M (2020). Venetoclax plus LDAC for newly diagnosed AML ineligible for intensive chemotherapy: a phase 3 randomized placebo-controlled trial. Blood.

[9] Shahswar R, Beutel G, Klement P, Rehberg A, Gabdoulline R, Koenecke C, Markel D, Eggers H, Eder M, Stadler M (2019). FLA-IDA salvage chemotherapy combined with a seven-day course of venetoclax (FLAVIDA) in patients with relapsed/refractory acute leukaemia. Br J Haematol.

[10] DiNardo CD, Albitar M, Kadia TM, Naqvi K, Vaughan K, Cavazos A, Pierce SA, Takahashi K, Kornblau SM, Ravandi F (2018). Venetoclax in combination with FLAG-IDA chemotherapy (FLAG-V-I) for fit, relapsed/refractory AML patients: interim results of a phase 1b/2 dose escalation and expansion study. Blood.

[11] Lachowiez C, Konopleva M, Kadia TM, Daver N, Loghavi S, Wang SA, Adeoti M, Pierce SA, Takahashi K, Short NJ (2020). Interim analysis of the phase 1b/2 study of the BCL-2 inhibitor venetoclax in combination with standard intensive AML induction/consolidation therapy with FLAG-IDA in patients with newly diagnosed or relapsed/refractory AML. Blood.

[12] Chua CC, Roberts AW, Reynolds J, Fong CY, Ting SB, Salmon JM, MacRaild S, Ivey A, Tiong IS, Fleming S (2020). Chemotherapy and venetoclax in elderly acute myeloid leukemia trial (CAVEAT): a phase Ib dose-escalation study of venetoclax combined with modified intensive chemotherapy. J Clin Oncol.

[13] Maiti A, Dinardo CD, Pemmaraju N, Kadia TM, Rausch CR, Naqvi K, Daver NG, Borthakur G, Ohanian M, Issa GC (2020). Ten-day decitabine with venetoclax (DEC10-VEN) in AML and high-risk (HR) MDS. J Clin Oncol.

[14] Kadia TM, Garcia-Manero G, Yilmaz M, Dinardo CD, Konopleva M, Montalban-Bravo G, Borthakur G, Jabbour E, Jain N, Andreeff M (2020). Venetoclax (Ven) added to intensive chemo with cladribine, idarubicin, and AraC (CLIA) achieves high rates of durable complete remission with low rates of measurable residual disease (MRD) in pts with newly diagnosed acute myeloid leukemia (AML). J Clin Oncol.

[15] Kadia TM, Borthakur G, Pemmaraju N, Daver N, DiNardo CD, Sasaki K, Issa GC, Ohanian M, Montalban Bravo G, Short NJ (2020). Phase II study of venetoclax added to cladribine + low dose AraC (LDAC) alternating with 5-azacytidine demonstrates high rates of minimal residual disease (MRD) negative complete remissions (CR) and excellent tolerability in older patients with newly diagnosed acute myeloid leukemia (AML). Blood.

[16] Kadia TM, Borthakur G, Takahashi K, DiNardo CD, Daver N, Pemmaraju N, Jabbour E, Jain N, Short NJ, Qiao W (2020). Phase II study of CPX-351 plus venetoclax in patients with acute myeloid leukemia (AML). Blood.

[17] Lin TL, Uy GL, Walter RB, Zou H, Wang Q, Faderl S, Cheung R, Pullarkat VA (2020). Phase Ib study of CPX-351 lower-intensity therapy (LIT) plus venetoclax as first-line treatment for patients with AML who are unfit for intensive chemotherapy (IC). J Clin Oncol.

[18] Arain S, Christian S, Patel PR, Sweiss K, Parkin B, Calip GS, Quigley JG (2020). Safety and efficacy of gemtuzumab ozogamicin and venetoclax in patients with relapsed or refractory CD33+ acute myeloid leukemia: a phase Ib study. J Clin Oncol.

[19] Maiti A, DiNardo CD, Kadia TM, Jabbour E, Rausch CR, Daver N, Borthakur G, Pemmaraju N, Short NJ, Yilmaz M (2020). Ten-day decitabine with venetoclax versus intensive chemotherapy in relapsed or refractory acute myeloid leukemia: a propensity score matched analysis. Blood.

[20] Lachowiez C, DiNardo CD, Konopleva M (2020). Venetoclax in acute myeloid leukemia-current and future directions. Leuk Lymphoma.

[21] Maiti A, Franquiz MJ, Ravandi F, Cortes JE, Jabbour EJ, Sasaki K, Marx K, Daver NG, Kadia TM, Konopleva MY (2020). Venetoclax and BCR-ABL tyrosine kinase inhibitor combinations: outcome in patients with philadelphia chromosome-positive advanced myeloid leukemias. Acta Haematol.

[22] Phillips DC, Jin S, Gregory GP, Zhang Q, Xue J, Zhao X, Chen J, Tong Y, Zhang H, Smith M (2019). A novel CDK9 inhibitor increases the efficacy of venetoclax (ABT-199) in multiple models of hematologic malignancies. Leukemia.

[23] Fischer MA, Friedlander SY, Arrate MP, Chang H, Gorska AE, Fuller LD, Ramsey HE, Kashyap T, Argueta C, Debler S (2020). Venetoclax response is enhanced by selective inhibitor of nuclear export compounds in hematologic malignancies. Blood Adv.

[24] Fiskus W, Cai T, DiNardo CD, Kornblau SM, Borthakur G, Kadia TM, Pemmaraju N, Bose P, Masarova L, Rajapakshe K (2019). Superior efficacy of cotreatment with BET protein inhibitor and BCL2 or MCL1 inhibitor against AML blast progenitor cells. Blood Cancer J.

[25] Elgamal OA, Mehmood A, Jeon JY, Carmichael B, Lehman A, Orwick SJ, Truxall J, Goettl VM, Wasmuth R, Tran M (2020). Preclinical efficacy for a novel tyrosine kinase inhibitor, ArQule 531 against acute myeloid leukemia. J Hematol Oncol.

[26] Maiti A, Rausch CR, Cortes JE, Pemmaraju N, Daver NG, Ravandi F, Garcia-Manero G, Borthakur G, Naqvi K, Ohanian M et al. Outcomes of relapsed or refractory acute myeloid leukemia after frontline hypomethylating agent and venetoclax regimens. Haematologica. 2020. 10.3324/haematol.2020.252569 PMC7927994 32499238

[27] DiNardo CD, Tiong IS, Quaglieri A, MacRaild S, Loghavi S, Brown FC, Thijssen R, Pomilio G, Ivey A, Salmon JM (2020). Molecular patterns of response and treatment failure after frontline venetoclax combinations in older patients with AML. Blood.

[28] Nechiporuk T, Kurtz SE, Nikolova O, Liu T, Jones CL, D'Alessandro A, Culp-Hill R, d'Almeida A, Joshi SK, Rosenberg M (2019). The TP53 apoptotic network is a primary mediator of resistance to BCL2 inhibition in AML cells. Cancer Discov.

[29] Pei S, Pollyea DA, Gustafson A, Stevens BM, Minhajuddin M, Fu R, Riemondy KA, Gillen AE, Sheridan RM, Kim J (2020). Monocytic subclones confer resistance to venetoclax-based therapy in patients with acute myeloid leukemia. Cancer Discov.

[30] Short NJ, Konopleva M, Kadia TM, Borthakur G, Ravandi F, DiNardo CD, Daver N (2020). Advances in the treatment of acute myeloid leukemia: new drugs and new challenges. Cancer Discov.

[31] DiNardo CD, Stein EM, de Botton S, Roboz GJ, Altman JK, Mims AS, Swords R, Collins RH, Mannis GN, Pollyea DA (2018). Durable remissions with ivosidenib in IDH1-mutated relapsed or refractory AML. N Engl J Med.

[32] Stein EM, DiNardo CD, Pollyea DA, Fathi AT, Roboz GJ, Altman JK, Stone RM, DeAngelo DJ, Levine RL, Flinn IW (2017). Enasidenib in mutant IDH2 relapsed or refractory acute myeloid leukemia. Blood.

[33] Pollyea DA, Tallman MS, de Botton S, Kantarjian HM, Collins R, Stein AS, Frattini MG, Xu Q, Tosolini A, See WL (2019). Enasidenib, an inhibitor of mutant IDH2 proteins, induces durable remissions in older patients with newly diagnosed acute myeloid leukemia. Leukemia.

[34] Roboz GJ, DiNardo CD, Stein EM, de Botton S, Mims AS, Prince GT, Altman JK, Arellano ML, Donnellan W, Erba HP (2019). Ivosidenib induces deep durable remissions in patients with newly diagnosed IDH1-mutant acute myeloid leukemia. Blood.

[35] Fathi AT, DiNardo CD, Kline I, Kenvin L, Gupta I, Attar EC, Stein EM, de Botton S (2018). Differentiation syndrome associated with enasidenib, a selective inhibitor of mutant isocitrate dehydrogenase 2: analysis of a phase 1/2 study. JAMA Oncol.

[36] Birendra KC, DiNardo CD (2016). Evidence for clinical differentiation and differentiation syndrome in patients with acute myeloid leukemia and IDH1 mutations treated with the targeted mutant IDH1 inhibitor, AG-120. Clin Lymphoma Myeloma Leuk.

[37] DiNardo CD, Propert KJ, Loren AW, Paietta E, Sun Z, Levine RL, Straley KS, Yen K, Patel JP, Agresta S (2013). Serum 2-hydroxyglutarate levels predict isocitrate dehydrogenase mutations and clinical outcome in acute myeloid leukemia. Blood.

[38] Quek L, David MD, Kennedy A, Metzner M, Amatangelo M, Shih A, Stoilova B, Quivoron C, Heiblig M, Willekens C (2018). Clonal heterogeneity of acute myeloid leukemia treated with the IDH2 inhibitor enasidenib. Nat Med.

[39] Harding JJ, Lowery MA, Shih AH, Schvartzman JM, Hou S, Famulare C, Patel M, Roshal M, Do RK, Zehir A (2018). Isoform switching as a mechanism of acquired resistance to mutant isocitrate dehydrogenase inhibition. Cancer Discov.

[40] Intlekofer AM, Shih AH, Wang B, Nazir A, Rustenburg AS, Albanese SK, Patel M, Famulare C, Correa FM, Takemoto N (2018). Acquired resistance to IDH inhibition through trans or cis dimer-interface mutations. Nature.

[41] Dinardo CD, Stein AS, Stein EM, Fathi AT, Frankfurt O, Schuh AC, Martinelli G, Patel PA, Raffoux E, Tan P (2019). Mutant IDH1 inhibitor ivosidenib (IVO; AG-120) in combination with azacitidine (AZA) for newly diagnosed acute myeloid leukemia (ND AML). J Clin Oncol.

[42] Daigle SR, Choe S, Quek L, DiNardo CD, Stein A, Stein EM, Fathi AT, Frankfurt O, Schuh AC, Kantarjian HM (2019). High rate of IDH1 mutation clearance and measurable residual disease negativity in patients with IDH1-mutant newly diagnosed acute myeloid leukemia treated with ivosidenib (AG-120) and azacitidine. Blood.

[43] Fernandez PM, Recher C, Doronin V, Calado RT, Jang JH, Miyazaki Y, Wang J, Gianolio DA, Daigle SR, Winkler T (2019). AGILE: a phase 3, multicenter, double-blind, randomized, placebo-controlled study of ivosidenib in combination with azacitidine in adult patients with previously untreated acute myeloid leukemia with an IDH1 mutation. Blood.

[44] Dinardo CD, Schuh AC, Stein EM, Montesinos P, Wei A, Botton SD, Zeidan AM, Fathi AT, Quek L, Kantarjian HM (2020). Effect of enasidenib (ENA) plus azacitidine (AZA) on complete remission and overall response versus AZA monotherapy in mutant-IDH2 (mIDH2) newly diagnosed acute myeloid leukemia (ND-AML). J Clin Oncol.

[45] Pollyea DA, Dinardo CD, Arellano ML, Pigneux A, Fiedler W, Konopleva M, Rizzieri DA, Smith BD, Shinagawa A, Lemoli RM (2020). Results of venetoclax and azacitidine combination in chemotherapy ineligible untreated patients with acute myeloid leukemia with IDH 1/2 mutations. Blood.

[46] Chan SM, Thomas D, Corces-Zimmerman MR, Xavy S, Rastogi S, Hong WJ, Zhao F, Medeiros BC, Tyvoll DA, Majeti R (2015). Isocitrate dehydrogenase 1 and 2 mutations induce BCL-2 dependence in acute myeloid leukemia. Nat Med.

[47] Lachowiez CA, Borthakur G, Loghavi S, Zeng Z, Kadia TM, Masarova L, Takahashi K, Tippett GD, Naqvi K, Bose P (2020). Phase Ib/II study of the IDH1-mutant inhibitor ivosidenib with the BCL2 inhibitor venetoclax +/- azacitidine in IDH1-mutated hematologic malignancies. J Clin Oncol.

[48] Cathelin S, Sharon D, Subedi A, Cojocari D, Phillips DC, Leverson JD, MacBeth K, Nicolay B, Narayanaswamy R, Ronseaux S (2018). Combination of enasidenib and venetoclax shows superior anti-leukemic activity against IDH2 mutated AML in patient-derived xenograft models. Blood.

[49] DiNardo CD, Stein AS, Stein EM, Fathi AT, Frankfurt O, Schuh AC, Döhner H, Martinelli G, Patel PA, Raffoux E (2021). Mutant isocitrate dehydrogenase 1 inhibitor ivosidenib in combination with azacitidine for newly diagnosed acute myeloid leukemia. J Clin Oncol.

[50] DiNardo CD, Schuh AC, Stein EM, Fernandez PM, Wei A, De Botton S, Zeidan AM, Fathi AT, Quek L, Kantarjian HM (2019). Enasidenib plus azacitidine significantly improves complete remission and overall response compared with azacitidine alone in patients with newly diagnosed acute myeloid leukemia (AML) with isocitrate dehydrogenase 2 (IDH2) mutations: interim phase II results from an ongoing, randomized study. Blood.

[51] Stone RM, Mandrekar SJ, Sanford BL, Laumann K, Geyer S, Bloomfield CD, Thiede C, Prior TW, Döhner K, Marcucci G (2017). Midostaurin plus chemotherapy for acute myeloid leukemia with a FLT3 mutation. N Engl J Med.

[52] Perl AE, Martinelli G, Cortes JE, Neubauer A, Berman E, Paolini S, Montesinos P, Baer MR, Larson RA, Ustun C (2019). Gilteritinib or chemotherapy for relapsed or refractory FLT3-mutated AML. N Engl J Med.

[53] Cortes JE, Khaled S, Martinelli G, Perl AE, Ganguly S, Russell N, Kramer A, Dombret H, Hogge D, Jonas BA (2019). Quizartinib versus salvage chemotherapy in relapsed or refractory FLT3-ITD acute myeloid leukaemia (QuANTUM-R): a multicentre, randomised, controlled, open-label, phase 3 trial. Lancet Oncol.

[54] Pratz KW, Cherry M, Altman JK, Cooper BW, Cruz JC, Jurcic JG, Levis M, Lin T, Perl AE, Podoltsev NA (2020). A phase 1 study of gilteritinib in combination with induction and consolidation chemotherapy in patients with newly diagnosed AML: final results. Blood.

[55] Wang ES, Montesinos P, Minden MD, Lee J-H, Heuser M, Naoe T, Chou W-C, Liu S, Wu R, Philipose N et al. Phase 3, multicenter, open-label study of gilteritinib, gilteritinib plus azacitidine, or azacitidine alone in newly diagnosed FLT3 mutated (FLT3mut+) acute myeloid leukemia (AML) patients ineligible for intensive induction chemotherapy. In: ASH 2020. 2020.

[56] Daver N, Altman JK, Maly J, Levis M, Ritchie E, Litzow M, McCloskey JK, Smith CC, Schiller GJ, Bradley T (2020). Efficacy and safety of venetoclax in combination with gilteritinib for relapsed/refractory FLT3-mutated acute myeloid leukemia in the expansion cohort of a phase 1b study. Blood.

[57] Yilmaz M, Kantarjian HM, Muftuoglu M, Kadia TM, Konopleva M, Borthakur G, DiNardo CD, Pemmaraju N, Short NJ, Alvarado Y (2020). Quizartinib with decitabine +/- venetoclax is highly active in patients (Pts) with FLT3-ITD mutated (mut) acute myeloid Leukemia (AML): clinical report and signaling cytof profiling from a phase IB/II trial. Blood.

[58] Short NJ, Rytting ME, Cortes JE (2018). Acute myeloid leukaemia. Lancet.

[59] Hunter AM, Sallman DA (2020). Targeting TP53 mutations in myelodysplastic syndromes. Hematol Oncol Clin North Am.

[60] Bernard E, Nannya Y, Hasserjian RP, Devlin SM, Tuechler H, Medina-Martinez JS, Yoshizato T, Shiozawa Y, Saiki R, Malcovati L et al. Implications of TP53 allelic state for genome stability, clinical presentation and outcomes in myelodysplastic syndromes. Nat Med. 2020. 10.1038/s41591-020-1008-z PMC8381722 32747829

[61] Welch JS, Petti AA, Miller CA, Fronick CC, O'Laughlin M, Fulton RS, Wilson RK, Baty JD, Duncavage EJ, Tandon B (2016). TP53 and decitabine in acute myeloid leukemia and myelodysplastic syndromes. N Engl J Med.

[62] Short NJ, Kantarjian HM, Loghavi S, Huang X, Qiao W, Borthakur G, Kadia TM, Daver N, Ohanian M, Dinardo CD (2018). Treatment with a 5-day versus a 10-day schedule of decitabine in older patients with newly diagnosed acute myeloid leukaemia: a randomised phase 2 trial. Lancet Haematol.

[63] Lehmann S, Bykov VJ, Ali D, Andren O, Cherif H, Tidefelt U, Uggla B, Yachnin J, Juliusson G, Moshfegh A (2012). Targeting p53 in vivo: a first-in-human study with p53-targeting compound APR-246 in refractory hematologic malignancies and prostate cancer. J Clin Oncol.

[64] Sallman DA, DeZern AE, Garcia-Manero G, Steensma DP, Roboz GJ, Sekeres MA, Cluzeau T, Sweet KL, McLemore AF, McGraw K (2019). Phase 2 results of APR-246 and azacitidine (AZA) in patients with TP53 mutant myelodysplastic syndromes (MDS) and oligoblastic acute myeloid leukemia (AML). Blood.

[65] Cluzeau T, Sebert M, Rahmé R, Cuzzubo S, Walter-Petrich A, Lehmann-Che J, Madeleine I, Peterlin P, Bève B, Attalah H et al. APR-246 combined with azacitidine (AZA) in PT53 mutated myelodysplastic syndrome (MDS) and cute myeloid leukemia (AML). A phase 2 study by the Groupe Francophone Des Myelodysplasies (GFM). In: Presented at: 2020 European Hematology Association Congress; June 11–21, 2020; Virtual Abstract S181: 2020; 2020.

[66] Vassilev LT (2007). MDM2 inhibitors for cancer therapy. Trends Mol Med.

[67] Kojima K, Konopleva M, Samudio IJ, Shikami M, Cabreira-Hansen M, McQueen T, Ruvolo V, Tsao T, Zeng Z, Vassilev LT (2005). MDM2 antagonists induce p53-dependent apoptosis in AML: implications for leukemia therapy. Blood.

[68] Khurana A, Shafer DA (2019). MDM2 antagonists as a novel treatment option for acute myeloid leukemia: perspectives on the therapeutic potential of idasanutlin (RG7388). Onco Targets Ther.

[69] Efeyan A, Ortega-Molina A, Velasco-Miguel S, Herranz D, Vassilev LT, Serrano M (2007). Induction of p53-dependent senescence by the MDM2 antagonist nutlin-3a in mouse cells of fibroblast origin. Cancer Res.

[70] Andreeff M, Kelly KR, Yee K, Assouline S, Strair R, Popplewell L, Bowen D, Martinelli G, Drummond MW, Vyas P (2015). Results of the phase I trial of RG7112, a small-molecule MDM2 antagonist in leukemia. Clin Cancer Res.

[71] Uy GL, Assouline S, Young AM, Blotner S, Higgins B, Chen LC, Yee K (2020). Phase 1 study of the MDM2 antagonist RO6839921 in patients with acute myeloid leukemia. Invest New Drugs.

[72] Erba HP, Becker PS, Shami PJ, Grunwald MR, Flesher DL, Zhu M, Rasmussen E, Henary HA, Anderson AA, Wang ES (2019). Phase 1b study of the MDM2 inhibitor AMG 232 with or without trametinib in relapsed/refractory acute myeloid leukemia. Blood Adv.

[73] Konopleva M, Martinelli G, Daver N, Papayannidis C, Wei A, Higgins B, Ott M, Mascarenhas J, Andreeff M. MDM2 inhibition: an important step forward in cancer therapy. Leukemia. 2020. 10.1038/s41375-020-0949-z 32651541

[74] Ishizawa J, Nakamaru K, Seki T, Tazaki K, Kojima K, Chachad D, Zhao R, Heese L, Ma W, Ma MCJ (2018). Predictive gene signatures determine tumor sensitivity to MDM2 inhibition. Cancer Res.

[75] Montesinos P, Beckermann BM, Catalani O, Esteve J, Gamel K, Konopleva MY, Martinelli G, Monnet A, Papayannidis C, Park A (2020). MIRROS: a randomized, placebo-controlled, phase III trial of cytarabine +/− idasanutlin in relapsed or refractory acute myeloid leukemia. Future Oncol.

[76] Thomas A, El Rouby S, Reed JC, Krajewski S, Silber R, Potmesil M, Newcomb EW (1996). Drug-induced apoptosis in B-cell chronic lymphocytic leukemia: relationship between p53 gene mutation and bcl-2/bax proteins in drug resistance. Oncogene.

[77] Perego P, Giarola M, Righetti SC, Supino R, Caserini C, Delia D, Pierotti MA, Miyashita T, Reed JC, Zunino F (1996). Association between cisplatin resistance and mutation of p53 gene and reduced bax expression in ovarian carcinoma cell systems. Cancer Res.

[78] Eliopoulos AG, Kerr DJ, Herod J, Hodgkins L, Krajewski S, Reed JC, Young LS (1995). The control of apoptosis and drug resistance in ovarian cancer: influence of p53 and Bcl-2. Oncogene.

[79] Miyashita T, Reed JC (1995). Tumor suppressor p53 is a direct transcriptional activator of the human bax gene. Cell.

[80] Miyashita T, Harigai M, Hanada M, Reed JC (1994). Identification of a p53-dependent negative response element in the bcl-2 gene. Cancer Res.

[81] Miyashita T, Krajewski S, Krajewska M, Wang HG, Lin HK, Liebermann DA, Hoffman B, Reed JC (1994). Tumor suppressor p53 is a regulator of bcl-2 and bax gene expression in vitro and in vivo. Oncogene.

[82] Tagscherer KE, Fassl A, Sinkovic T, Combs SE, Roth W (2011). p53-dependent regulation of Mcl-1 contributes to synergistic cell death by ionizing radiation and the Bcl-2/Bcl-XL inhibitor ABT-737. Apoptosis.

[83] Carter BZ, Mak DH, Schober WD, Koller E, Pinilla C, Vassilev LT, Reed JC, Andreeff M (2009). Simultaneous activation of p53 and inhibition of XIAP enhance the activation of apoptosis signaling pathways in AML. Blood.

[84] Pan R, Ruvolo V, Mu H, Leverson JD, Nichols G, Reed JC, Konopleva M, Andreeff M (2017). Synthetic Lethality of combined Bcl-2 Inhibition and p53 activation in AML: mechanisms and superior antileukemic efficacy. Cancer Cell.

[85] Daver NG, Pollyea DA, Garcia JS, Jonas BA, Yee KWL, Fenaux P, Assouline S, Vey N, Olin R, Roboz GJ (2018). Safety, efficacy, pharmacokinetic (PK) and biomarker analyses of BCL2 inhibitor venetoclax (Ven) Plus MDM2 inhibitor idasanutlin (idasa) in patients (pts) with relapsed or refractory (R/R) AML: a phase Ib, non-randomized, open-label study. Blood.

[86] Davids MS, Kim HT, Bachireddy P, Costello C, Liguori R, Savell A, Lukez AP, Avigan D, Chen YB, McSweeney P (2016). Ipilimumab for patients with relapse after allogeneic transplantation. N Engl J Med.

[87] Daver N, Garcia-Manero G, Basu S, Boddu PC, Alfayez M, Cortes JE, Konopleva M, Ravandi-Kashani F, Jabbour E, Kadia T (2018). Efficacy, safety, and biomarkers of response to azacitidine and nivolumab in relapsed/refractory acute myeloid leukemia: a nonrandomized, open-label, phase II study. Cancer Discov.

[88] Zeidan AM, Cavenagh J, Voso MT, Taussig D, Tormo M, Boss I, Copeland WB, Gray VE, Previtali A, O'Connor T (2019). Efficacy and safety of azacitidine (AZA) in combination with the anti-PD-L1 durvalumab (durva) for the front-line treatment of older patients (pts) with acute myeloid leukemia (AML) who are unfit for intensive chemotherapy (IC) and Pts with higher-risk myelodysplastic syndromes (HR-MDS): results from a large, international, randomized phase 2 study. Blood.

[89] Kadia TM, Cortes JE, Ghorab A, Ravandi F, Jabbour E, Daver NG, Alvarado Y, Ohanian M, Konopleva M, Kantarjian HM (2018). Nivolumab (Nivo) maintenance (maint) in high-risk (HR) acute myeloid leukemia (AML) patients. J Clin Oncol.

[90] Sallman DA, Asch AS, Kambhampati S, Al Malki MM, Zeidner JF, Donnellan W, Lee DJ, Vyas P, Jeyakumar D, Mannis GN et al. The first-in-class anti-CD47 antibody magrolimab combined with azacitidine is well-tolerated and effective in AML patients: phase 1b results. In: ASH 2020, Abstract 330. 2020.

[91] Borate U, Esteve J, Porkka K, Knapper S, Vey N, Scholl S, Garcia-Manero G, Wermke M, Janssen J, Traer E et al. Anti-TIM-3 antibody MBG453 in combination with hypomethylating agents in patients with high-risk myelodysplastic syndrome and acute myeloid leukemia: a phase 1 study. In: Abstract presented at: the 25th European Hematology Association Congress; June 11–21, 2020 Abstract S185. 2020.

[92] Daver NG, Montesinos P, DeAngelo DJ, Wang ES, Papadantonakis N, Deconinck E, Erba HP, Pemmaraju N, Lane AA, Rizzieri DA (2019). Clinical profile of IMGN632, a novel CD123-targeting antibody-drug conjugate (ADC), in patients with relapsed/refractory (R/R) acute myeloid leukemia (AML) or blastic plasmacytoid dendritic cell neoplasm (BPDCN). Blood.

[93] Aldoss I, Uy GL, Vey N, Emadi A, Sayre PH, Walter RB, Foster MC, Arellano ML, Godwin JE, Wieduwilt MJ (2020). Flotetuzumab as salvage therapy for primary induction failure and early relapse acute myeloid leukemia. Blood.

[94] Ravandi F, Bashey A, Stock W, Foran JM, Mawad R, Egan D, Blum W, Yang A, Pastore A, Johnson C (2020). Complete responses in relapsed/refractory acute myeloid leukemia (AML) patients on a weekly dosing schedule of vibecotamab (XmAb14045), a CD123 x CD3 T cell-engaging bispecific antibody; initial results of a phase 1 study. Blood.

[95] Zhou Q, Munger ME, Veenstra RG, Weigel BJ, Hirashima M, Munn DH, Murphy WJ, Azuma M, Anderson AC, Kuchroo VK (2011). Coexpression of Tim-3 and PD-1 identifies a CD8+ T-cell exhaustion phenotype in mice with disseminated acute myelogenous leukemia. Blood.

[96] Zhang L, Gajewski TF, Kline J (2009). PD-1/PD-L1 interactions inhibit antitumor immune responses in a murine acute myeloid leukemia model. Blood.

[97] Zhou Q, Munger ME, Highfill SL, Tolar J, Weigel BJ, Riddle M, Sharpe AH, Vallera DA, Azuma M, Levine BL (2010). Program death-1 signaling and regulatory T cells collaborate to resist the function of adoptively transferred cytotoxic T lymphocytes in advanced acute myeloid leukemia. Blood.

[98] Williams P, Basu S, Garcia-Manero G, Hourigan CS, Oetjen KA, Cortes JE, Ravandi F, Jabbour EJ, Al-Hamal Z, Konopleva M (2018). The distribution of T-cell subsets and the expression of immune checkpoint receptors and ligands in patients with newly diagnosed and relapsed acute myeloid leukemia. Cancer.

[99] Yang H, Bueso-Ramos C, DiNardo C, Estecio MR, Davanlou M, Geng QR, Fang Z, Nguyen M, Pierce S, Wei Y (2013). Expression of PD-L1, PD-L2, PD-1 and CTLA4 in myelodysplastic syndromes is enhanced by treatment with hypomethylating agents. Leukemia.

[100] Dama P, Tang M, Fulton N, Kline J, Liu H (2019). Gal9/Tim-3 expression level is higher in AML patients who fail chemotherapy. J Immunother Cancer.

[101] Hay AE, Assouline S, Walter RB, Little RF, Moseley A, Gail SM, Im A, Foran JM, Radich JP, Fang M (2019). Accrual barriers and detection of early toxicity signal in older less-fit patients treated with azacitidine and nivolumab for newly diagnosed acute myeloid leukemia (AML) or high-risk myelodysplastic syndrome (MDS) in the SWOG 1612 platform randomized phase II/III clinical trial. Blood.

[102] Chao MP, Takimoto CH, Feng DD, McKenna K, Gip P, Liu J, Volkmer JP, Weissman IL, Majeti R (2020). Therapeutic targeting of the macrophage immune checkpoint CD47 in myeloid malignancies. Front Oncol.

[103] Sallman DA, Asch AS, Al Malki MM, Lee DJ, Donnellan WB, Marcucci G, Kambhampati S, Daver NG, Garcia-Manero G, Komrokji RS (2019). The first-in-class anti-CD47 antibody magrolimab (5F9) in combination with azacitidine is effective in MDS and AML patients: ongoing phase 1b results. Blood.

[104] Wolf Y, Anderson AC, Kuchroo VK (2019). TIM3 comes of age as an inhibitory receptor. Nat Rev Immunol.

[105] Kikushige Y, Shima T, Takayanagi S, Urata S, Miyamoto T, Iwasaki H, Takenaka K, Teshima T, Tanaka T, Inagaki Y (2010). TIM-3 is a promising target to selectively kill acute myeloid leukemia stem cells. Cell Stem Cell.

[106] Borate U, Esteve J, Porkka K, Knapper S, Vey N, Scholl S, Garcia-Manero G, Wermke M, Janssen J, Traer E (2019). Phase Ib study of the anti-TIM-3 antibody MBG453 in combination with decitabine in patients with high-risk myelodysplastic syndrome (MDS) and acute myeloid leukemia (AML). Blood.

[107] Brunner AM, Esteve J, Porkka K, Knapper S, Vey N, Scholl S, Garcia-Manero G, Wermke M, Janssen J, Traer E (2020). Efficacy and safety of sabatolimab (MBG453) in combination with hypomethylating agents (HMAs) in patients with acute myeloid leukemia (AML) and high-risk myelodysplastic syndrome (HR-MDS): updated results from a phase 1b study. Blood.

[108] Wang ES, Adés L, Fathi AT, Kreuzer KA, O'Meara MM, Liang S-Y, Ravandi F (2017). CASCADE: a phase 3, randomized, double-blind study of vadastuximab talirine (33A) versus placebo in combination with azacitidine or decitabine in the treatment of older patients with newly diagnosed acute myeloid leukemia (AML). J Clin Oncol.

[109] Nair-Gupta P, Diem M, Reeves D, Wang W, Schulingkamp R, Sproesser K, Mattson B, Heidrich B, Mendonça M, Joseph J (2019). A novel C2 domain binding CD33xCD3 bispecific antibody with potent T-cell redirection activity against acute myeloid leukemia. Blood Adv.

[110] Pemmaraju N, Lane AA, Sweet KL, Stein AS, Vasu S, Blum W, Rizzieri DA, Wang ES, Duvic M, Sloan JM (2019). Tagraxofusp in blastic plasmacytoid dendritic-cell neoplasm. N Engl J Med.

[111] Kovtun Y, Jones GE, Adams S, Harvey L, Audette CA, Wilhelm A, Bai C, Rui L, Laleau R, Liu F (2018). A CD123-targeting antibody-drug conjugate, IMGN632, designed to eradicate AML while sparing normal bone marrow cells. Blood Adv.

[112] Daver NG, Erba HP, Papadantonakis N, DeAngelo DJ, Wang ES, Konopleva MY, Sloss CM, Wang J, Malcolm KE, Zweidler-McKay PA (2019). A phase 1b/2 study of the CD123-targeting antibody-drug conjugate IMGN632 as monotherapy or in combination with venetoclax and/or azacitidine for patients with CD123-positive acute myeloid leukemia. Blood.

[113] Adams S, Zhang Q, McCarthy R, Flaherty LJ, Watkins K, Sloss CM, Romanelli A, Zweidler-McKay P, Konopleva M (2020). The combination OF IMGN632, a CD123-targeting ADC, with venetoclax enhances anti-leukemic activity in vitro and prolongs survival in vivo in pre-clinical models of human AML. HemaSphere.

[114] Chichili GR, Huang L, Li H, Burke S, He L, Tang Q, Jin L, Gorlatov S, Ciccarone V, Chen F (2015). A CD3xCD123 bispecific DART for redirecting host T cells to myelogenous leukemia: preclinical activity and safety in nonhuman primates. Sci Transl Med.

[115] Uy GL, Aldoss I, Foster MC, Sayre PH, Wieduwilt MJ, Advani AS, Godwin JE, Arellano ML, Sweet KL, Emadi A (2021). Flotetuzumab as salvage immunotherapy for refractory acute myeloid leukemia. Blood.

[116] Ravandi F, Bashey A, Foran JM, Stock W, Mawad R, Blum W, Saville MW, Johnson CM, Vanasse KGJ, Ly T (2018). Complete responses in relapsed/refractory acute myeloid leukemia (AML) patients on a weekly dosing schedule of XmAb14045, a CD123 x CD3 T cell-engaging bispecific antibody: initial results of a phase 1 study. Blood.

[117] Braciak TA, Roskopf CC, Wildenhain S, Fenn NC, Schiller CB, Schubert IA, Jacob U, Honegger A, Krupka C, Subklewe M et al. Dual-targeting triplebody 33-16-123 (SPM-2) mediates effective redirected lysis of primary blasts from patients with a broad range of AML subtypes in combination with natural killer cells. In: Oncoimmunology. vol. 7. Taylor & Francis; 2018: e1472195. 10.1080/2162402X.2018.1472195 PMC6140553 30228941

[118] Ma H, Padmanabhan IS, Parmar S, Gong Y (2019). Targeting CLL-1 for acute myeloid leukemia therapy. J Hematol Oncol.

[119] Hofmann S, Schubert M-L, Wang L, He B, Neuber B, Dreger P, Maller-Tidow C, Schmitt M (2019). Chimeric antigen receptor (CAR) T cell therapy in acute myeloid leukemia (AML). J Clin Med.

[120] John S, Chen H, Deng M, Gui X, Wu G, Chen W, Li Z, Zhang N, An Z, Zhang CC (2018). A novel anti-LILRB4 CAR-T cell for the treatment of monocytic AML. Mol Ther.

[121] Perales MA, Kebriaei P, Kean LS, Sadelain M (2018). Building a safer and faster CAR: seatbelts, airbags, and CRISPR. Biol Blood Marrow Transplant.

[122] Wang QS, Wang Y, Lv HY, Han QW, Fan H, Guo B, Wang LL, Han WD (2015). Treatment of CD33-directed chimeric antigen receptor-modified T cells in one patient with relapsed and refractory acute myeloid leukemia. Mol Ther.

[123] Liu F, Cao Y, Pinz K, Ma Y, Wada M, Chen K, Ma G, Shen J, Tse CO, Su Y (2018). First-in-human CLL1-CD33 compound CAR T cell therapy induces complete remission in patients with refractory acute myeloid leukemia: update on phase 1 clinical trial. Blood.

[124] Liu F, Zhang H, Sun L, Li Y, Zhang S, He G, Yi H, Wada M, Pinz KG, Chen KH et al. First-in-human CLL1-CD33 compound CAR (cCAR) T cell therapy in relapsed and refractory acute myeloid leukemia. In: EHA 2020. vol. EHA Library. Liu F. 06/12/20; 294969; S149; 2020.

[125] Katherine DC, Saar G (2020). Chimeric antigen receptor T-cell therapy for acute myeloid leukemia: how close to reality?. Haematologica.

[126] Testa U, Pelosi E, Castelli G (2019). CD123 as a therapeutic target in the treatment of hematological malignancies. Cancers.

[127] Mardiros A, Dos Santos C, McDonald T, Brown CE, Wang X, Budde LE, Hoffman L, Aguilar B, Chang W-C, Bretzlaff W (2012). T cells expressing CD123-specific chimeric antigen receptors exhibit specific cytolytic effector functions and antitumor effects against human acute myeloid leukemia. Blood.

[128] Gill S, Tasian SK, Ruella M, Shestova O, Li Y, Porter DL, Carroll M, Danet-Desnoyers G, Scholler J, Grupp SA (2013). Preclinical targeting of human acute myeloid leukemia and myeloablation using chimeric antigen receptor-modified T cells. Blood.

[129] Burchert A, Bug G, Fritz LV, Finke J, Stelljes M, Rallig C, Wollmer E, Wasch R, Bornhauser M, Berg T (2020). Sorafenib maintenance after allogeneic hematopoietic stem cell transplantation for acute myeloid leukemia with FLT3-internal tandem duplication mutation (SORMAIN). J Clin Oncol.

[130] Wei AH, Dahner H, Pocock C, Montesinos P, Afanasyev B, Dombret H, Ravandi F, Sayar H, Jang JH, Porkka K (2019). The QUAZAR AML-001 maintenance trial: results of a phase III international, randomized, double-blind, placebo-controlled study of CC-486 (oral formulation of azacitidine) in patients with acute myeloid leukemia (AML) in first remission. Blood.

[131] Wei A, Roboz GJ, Dombret H, Dahner H, Schuh AC, Montesinos P, Pocock C, Selleslag D, Bondarenko SN, La Torre I (2020). CC-486 improves overall survival (OS) and relapse-free survival (RFS) for patients with acute myeloid leukemia (AML) in first remission after intensive chemotherapy (IC), regardless of amount of consolidation received: results from the phase III QUAZAR AML-001 maintenance trial. Blood.

[132] Roboz GJ, Ravandi F, Wei AH, Dombret H, Dahner H, Thol F, Voso MT, Schuh AC, Porkka K, La Torre I (2020). CC-486 prolongs survival for patients with acute myeloid leukemia (AML) in remission after intensive chemotherapy (IC) independent of the presence of measurable residual disease (MRD) at study entry: results from the QUAZAR AML-001 maintenance trial. Blood.

[133] Roboz GJ, Döhner H, Pocock C, Dombret H, Ravandi F, Jang JH, Selleslag D, Mayer J, Martens UM, Liesveld JL et al. Health-related quality of life with CC-486 in patients with acute myeloid leukemia (AML) in first remission following induction chemotherapy (IC): results from the phase III QUAZAR AML-001 maintenance trial. In: 62th ASH 2020 meeting. vol. Abstract 214; 2020.

[134] Dohner H, Wei A, Montesinos P, Dombret H, Ravandi F, Sayar H, Porkka K, Sandhu I, Passamonti F, Pane F (2020). Escalated dosing schedules of CC-486 for patients experiencing first acute myeloid leukemia (AML) relapse: Results from the phase III QUAZAR AML-001 maintenance trial. J Clin Oncol.

